# Caseinolytic Proteins (Clp) in the Genus *Klebsiella*: Special Focus on ClpK

**DOI:** 10.3390/molecules27010200

**Published:** 2021-12-29

**Authors:** Tehrim Motiwala, Blessing Oluebube Akumadu, Sbahle Zuma, Mbalenhle Sizamile Mfeka, Wanping Chen, Ikechukwu Achilonu, Khajamohiddin Syed, Thandeka Khoza

**Affiliations:** 1 Department of Biochemistry, School of Life Sciences, Pietermaritzburg Campus, University of Kwa-Zulu Natal, Pietermaritzburg, Scottsville 3209, South Africa; 216013319@stu.ukzn.ac.za (T.M.); ZumaS7@ukzn.ac.za (S.Z.); smmfeka850@gmail.com (M.S.M.); 2 Protein Structure-Function Research Unit, School of Molecular and Cell Biology, University of the Witwatersrand, Johannesburg 2050, South Africa; 1497819@students.wits.ac.za (B.O.A.); Ikechukwu.Achilonu@wits.ac.za (I.A.); 3 College of Food Science and Technology, Huazhong Agricultural University, Wuhan 430070, China; chenwanping1@foxmail.com; 4 Department of Biochemistry and Microbiology, Faculty of Science and Agriculture, University of Zululand, KwaDlangezwa 3886, South Africa

**Keywords:** *Klebsiella pneumoniae*, caseinolytic proteins, ClpK, antibiotic resistance, bioinformatics, expression, purification, molecular dynamic simulations, Far-UV CD, fluorescence spectroscopy, ATPase assay

## Abstract

Caseinolytic proteins (Clp), which are present in both prokaryotes and eukaryotes, play a major role in cell protein quality control and survival of bacteria in harsh environmental conditions. Recently, a member of this protein family, ClpK was identified in a pathogenic strain of *Klebsiella pneumoniae* which was responsible for nosocomial infections. ClpK is linked to the thermal stress survival of this pathogen. The genome wide analysis of Clp proteins in *Klebsiella* spp. indicates that ClpK is present in only 34% of the investigated strains. This suggests that the uptake of the *clpk* gene is selective and may only be taken up by a pathogen that needs to survive harsh environmental conditions. In silico analyses and molecular dynamic simulations show that ClpK is mainly α-helical and is highly dynamic. ClpK was successfully expressed and purified to homogeneity using affinity and anion exchange chromatography. Biophysical characterization of ClpK showed that it is predominantly alpha-helical, and this is in agreement with in silico analysis of the protein structure. Furthermore, the purified protein is biologically active and hydrolyses ATP in a concentration- dependent manner.

## 1. Introduction

*Klebsiella pneumoniae* are multi-drug resistant, aerobic, Gram-negative bacteria which belong to the *Enterobacteriaceae* family and were first isolated and described by Carl Friedlander in 1882 [1,2]. This opportunistic pathogen causes respiratory and urinary tract infections and has also been recognized as the cause of bloodstream infections in immunocompromised patients [2]. Furthermore, *Klebsiella* species have been found to be amongst the top three leading causes of hospital-acquired infections in the United States, mainly infecting immunocompromised individuals [2,3,4]. The infections caused by this pathogen are either endogenous or acquired through contact with an infected host or through contact with contaminated hospital equipment such as endoscopes [1]. Several members of the *Klebsiella* species have been found to be resistant to antibiotics such as amino- and carboxypenicillins [2,5,6,7]. Therefore, the *Klebsiella* species pose a serious threat especially to immunocompromised patients in hospital environments [2,5,6,7]. As a result, there is a constant drive to find alternative strategies to eliminate the threat posed by these organisms [8].

*K. pneumoniae* have developed various mechanisms to survive harsh environmental conditions [9]. One of these mechanisms includes the use of caseinolytic proteins (Clp), which play a role in maintaining protein homeostasis to adapt to changes in both the external and internal environment [10,11,12]. Clp proteins belong to the AAA+ (ATPases associated with diverse cellular activities) superfamily, and function as a complex of two components, namely the peptidase subunit (ClpP) and the regulatory subunits (ATPases) [9,10,13,14]. ClpK is amongst the recently identified ATPases. Other ATPases include ClpA, ClpB, ClpC and ClpX [10,11]. These ATPases are members of the Hsp100 family and promote the resolubilisation of proteins [10,11]. 

**Figure 1:**
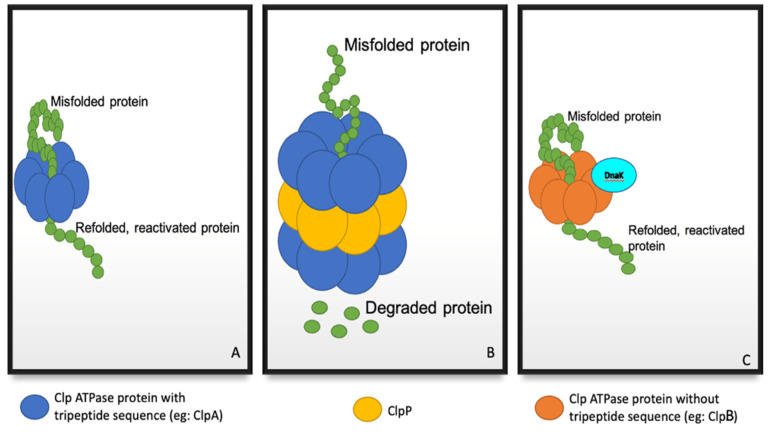
The function of the regulatory and catalytic components of Clp proteins. (**A**) The ATPase regulatory subunit (e.g., ClpA) alone, functions to unfold and reactivate misfolded proteins. (**B**) The ATPase regulatory and catalytic (ClpP) subunits function as a complex to degrade misfolded proteins which cannot be reactivated. (**C**) Some ATPase regulatory subunits (e.g., ClpB) do not interact with ClpP [10,11].

To maintain protein homeostasis under stressful conditions, misfolded proteins are recruited via regulatory subunits, unfolded, and reactivated using energy generated from ATP hydrolysis (Figure 1A) [10,11]. Proteins which are not successfully reactivated are then translocated into ClpP for degradation (Figure 1B) [10,11]. Certain ATPase regulatory subunits, such as ClpB, lack the tripeptide required for ClpP interaction, and therefore reactivate aggregated proteins through the assistance of chaperone systems such as the DnaK system (Figure 1C) [14].

To date, 12 Clp regulatory subunits with diverse cellular functions have been identified across various bacterial species (Table 1). These subunits are classified into either Class I or Class II depending on the number of nucleotide binding domains (NBDs) they contain. Class I and Class II members contain two and one NBDs, respectively [11,15,16]. Proteins belonging to class I are relatively large, and their sizes range from 68 kDa to 110 kDa, in contrast, proteins belonging to class II are considerably smaller [17]. The NBDs in Class I both consist of Domain 1 (D1) and Domain 2 (D2) however, the amino acid sequence of each domain differs. The difference in the amino acid sequence in the NBDs suggest that gene fusion, rather than gene duplication, may be the route through which members of class I evolved [14,18]. The NBDs contain canonical Walker A and Walker B which are essential for the breakdown of ATP. Walker A forms the floor of the nucleotide-binding pocket and binds the ATP phosphate. Walker B positions cations to bind metals that play a role in ATP catalysis [14,16,17,19].

Clp proteins are emerging as drug targets for various diseases caused by pathogens such as *Staphylococcus aureus*, *Streptococcus pneumoniae*, *Mycobacterium tuberculosis* and *Bacillus subtilis* due to their role in pathogen survival and pathogenicity [10,11,12,16,26]. It is therefore important to understand the diversity and functions of these proteins to adequately target their pathogenic nature and their survival in diverse environments. The well-studied Clp proteins in Class 1 include ClpA, ClpB and ClpC. However, very little is known about ClpK in terms of protein characteristics. In this study, a bioinformatics approach was used to investigate the presence of Clp regulatory subunits across the *Klebsiella* species, with a special focus on the presence of ClpK. We further performed in silico studies on the modelled structure of ClpK to gain insight into its structural features. To pave a way for functional and structural studies, we report ClpK expression and purification conditions. 

## 2. Results and Discussion

### 2.1. Caseinolytic ATPase Protein Classification

**Figure 2:**
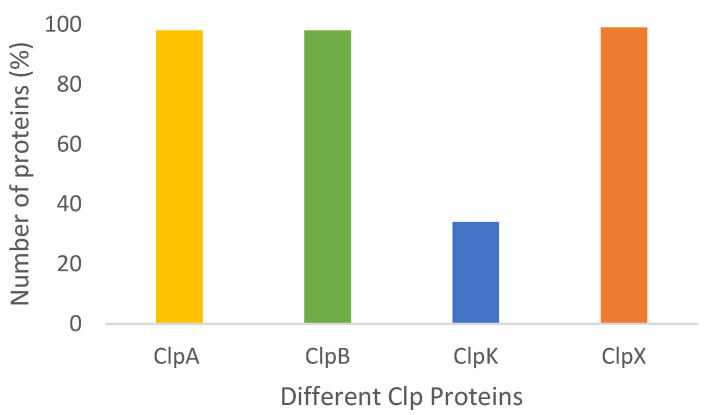
Clp proteins identified in the investigated *Klebsiella* strains. The total number of Clp proteins obtained from the National Center for Biotechnology Information (NCBI) Genome database, was tallied.

**Figure 3:**
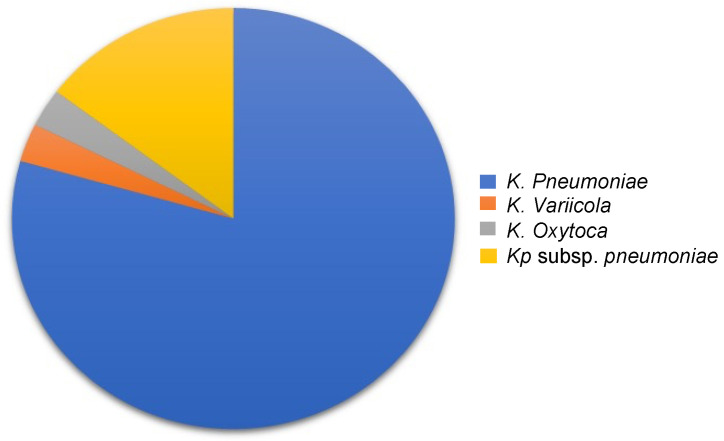
The distribution of ClpK proteins found among the investigated *Klebsiella* species. The total number of ClpK was tallied from data obtained using the NCBI Genome database. The highest number of ClpK was found in *K. pneumoniae* (27 strains), followed by *Kp* subsp. *pneumoniae* (5 strains), *K. variicola* (1 strain) and *K. oxytoca* (1 strain).

The presence and diversity of Clp regulatory subunits is a continuously studied field in different organisms; however, the presence of Clp proteins in the *Klebsiella* species has not been studied adequately [14,18,21,22,24,27]. To address this knowledge gap, the presence and diversity of Clp proteins in *Klebsiella* strains was investigated. It was observed that 98% of the strains studied contained ClpA, with the exception of *K. oxytoca* CAV1335 and *K. variicola* KP5-1 (Figure 2). A similar observation was noted for ClpB, where out of the studied species, only two (*K. variicola* KP5-1 and *K. variicola* DX120-E) did not contain ClpB (Figure 2). With respect to ClpX, *K. pneumoniae* CAV1217 was the only species that lacked the ClpX gene (Figure 2). Interestingly, we further observed that unlike ClpA, ClpB and ClpX, ClpK was only found to be present in 34% of the studied species. Additionally, only four out of the seven *Klebsiella* species contained ClpK with the highest number of ClpK was found within the *K. pneumoniae* species (Figure 3).

Our data shows a considerably low number of ClpK identified among the *Klebsiella* strains compared to the number of ClpA, ClpB and ClpX proteins. Out of the 57 *K. pneumoniae* strains which were analyzed, only 27 (47%) contained ClpK (Figure 2 and Figure 3). This was unexpected because ClpK is reported to be ubiquitous and is found in various species such as *Escherichia coli*, *Enterobacter cloacae*, and other *Klebsiella* strains other than *Klebsiella pneumoniae* [9]. Also, taking into account that the *clpk* gene is hypothesized to be transferred through horizontal gene transfer, one would expect it to be present among a greater number of the investigated strains [9]. However, the absence of ClpK in a majority of the studied strains may indicate the selective uptake of the *clpk* gene by pathogens to enable them to survive its current harsh environment. Our findings are similar to those obtained by Bojer, et al. (2010), wherein they reported that only 31 out of the 105 clinical isolates contained the *clpk* gene, suggesting that only certain strains acquired the plasmid through horizontal transfer to subsequently express the ClpK protein [9]. Interestingly, we also observed that none of the studied species contained ClpC. This observation correlates with the findings of Miller et al. (2018), who suggested that ClpC is a common ancestorial protein of ClpK and ClpA. Therefore inferring that the investigated strains may have contained ClpC at some point, which have now mutated into either ClpA or ClpK depending on environmental conditions [15].

### 2.2. Phylogenetic Analysis

A Clustal and phylogenetic tree analysis was performed to establish the relationship between the investigated *Klebsiella* Clp proteins. Phylogenetic tree analysis is a useful technique as it allows for representation of hierarchical of biological data and shows evolutionary relationships between species and how they have evolved over time [28]. Clustal analysis of the studies proteins was performed to evaluate the percentage identity shared within each group (Table 2). The percentage identity gives an estimation of the percentage residues that match up amongst proteins of interest [29]. Table 2 shows the high percentage identity of the compared protein sequences and therefore indicates that these are orthologs [30].

**Figure 4:**
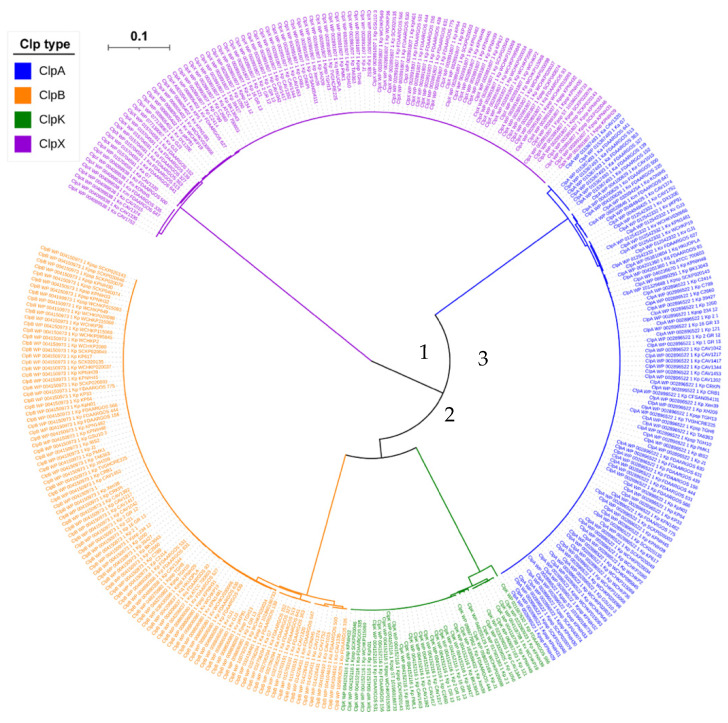
Phylogenetic tree of Clp proteins found among the seven Klebsiella species. MAFFT embedded in Trex servers was used to align the tree. Different Clp proteins are indicated in different colors. Centre of tree (1); point of ClpK and ClpB divergence (2); ClpA (3). Tree distance scale: 0.1.

The phylogenetic tree of the investigated Clp proteins is shown in Figure 4. The root of the phylogenetic tree is in the middle with branches radiating out in different directions. The branch of ClpX irradiates directly from the root which is expected because it belongs to Class II, whereas the other three proteins (ClpA, ClpB and ClpK) belong to Class I [17,20,21,23]. The branches of ClpA, ClpB and ClpK connect before divergence, and this suggests that these proteins have all descended from a common ancestor and are therefore termed to be homologs [30]. 

Figure 4 also shows divergence within the arrangement of the ClpK proteins. Clustal analysis of ClpK proteins namely, *K. pneumoniae* FDAARGOS 566, *K. pneumoniae* KPNIH39, *K. pneumoniae* 2-1, *K. pneumoniae* WCHKP020098, *K. pneumoniae* J1, *K. pneumoniae* FDAARGOS 444, *K. pneumoniae* CAV1417, and *Kp*. subsp. *pneumoniae* KPNIH32 from the various branches, showed an identity of 93.48–100%. The varying percentage identity and the visible divergence within ClpK indicates a divergence in the evolution of the protein [15].

### 2.3. Hypothetical ClpK Structure 

**Figure 5:**
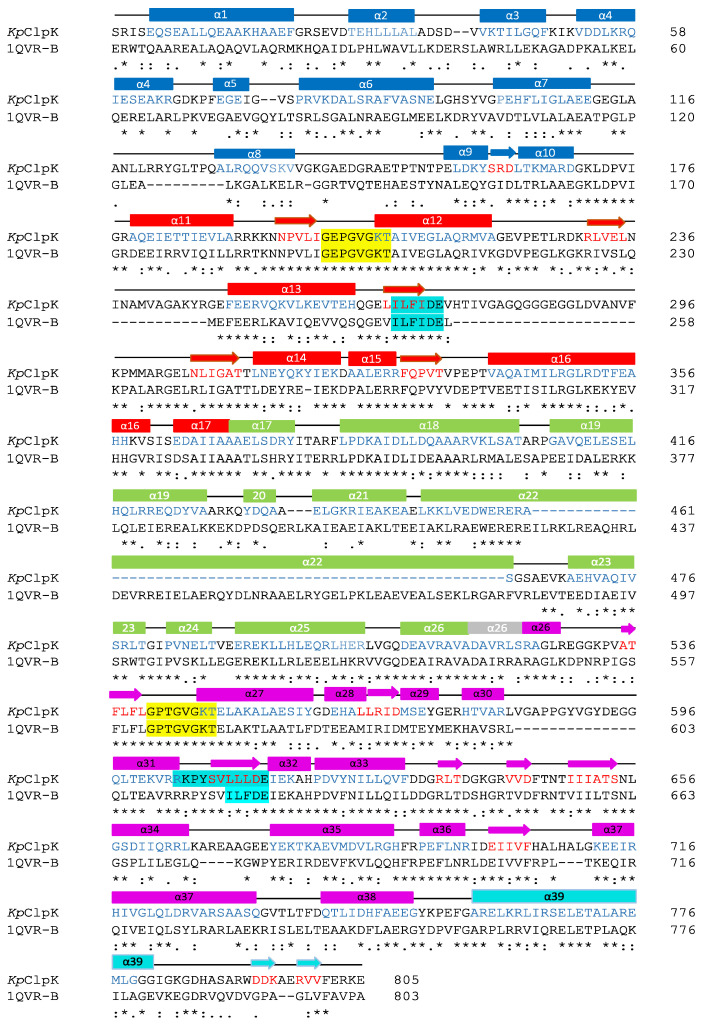
Alignment of ClpK with ClpB. The annotations were done according to sequence ClpB (1QVR-B). The α-helices and β-sheet are shown as rectangles and arrows, respectively. ClpK residues colored blue represent the position of the α-helices whereas red residues represent the position of the β-sheet. Asterisks (*) denote the identical residues, (:) represents sequence homologies, and (.) represents weak similarity. The five domains are shown as follows: N-terminal domain (blue); D1 large domain (red); D1 small domain (green); short linker region (grey); D2 large domain (pink); D2 small domain/C-terminal domain (cyan). The secondary structure elements are colored according to the domain. Walker A and Walker B motifs are highlighted yellow and cyan, respectively. The alignment was performed using T-COFFEE [32].

ClpB (1QVR-B) was identified as an appropriate template to model the structure of ClpK since it had a sequence identity and coverage query of 52% and 83%, respectively (Figure 5). The percentage query coverage indicates how much of the query sequence is included in the alignment; the higher the query coverage, the better the match [29]. Furthermore, the structure of ClpB was determined at 3.00 Å, and this was the best resolution compared to the resolution of other structures with similar percentage identity. This resolution is considered to be fairly good, as it allows for the visualization of well-defined water molecules and provides a fairly good idea about the shape of the macromolecule [31]. 

The alignment of ClpB and ClpK shows five domains namely, the N-terminal domain, D1-large domain, D1-small domain, D2-large domain and D2-small domain all of which are conserved within Class I proteins (Figure 5). The Walker A motif (GXXXGK[T/S]-X represents any residue) binds to ATP [14,15,16] and is 100% identical in the aligned sequences, suggesting that both these proteins interact with ATP in a similar manner. The Walker B motif (hhhhD[D/E]-h represents hydrophobic residues) binds metals, thus playing a role in ATP hydrolysis [14,15,16], and is 100% identical in NBD1 of the aligned sequence, while it only has 73% identity in NBD2. The non-identical residues in NBD2 may indicate that there are subtle differences in the binding of metals between these proteins.

**Figure 6:**
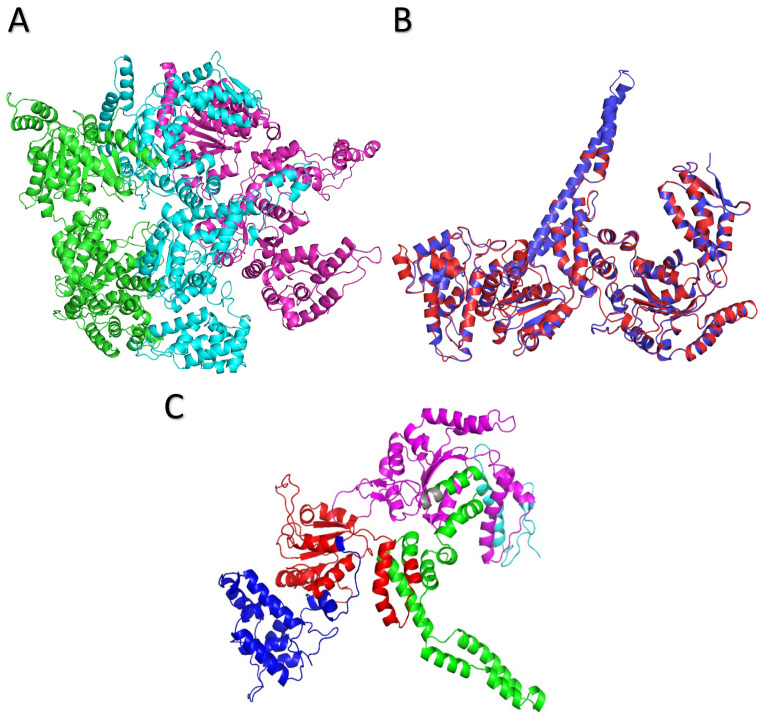
Hypothetical ClpK structure modelled using ClpB as a template. (**A**) Secondary trimeric structure of ClpK with chains A, B, and C shown as green, cyan and pink, respectively. (**B**) Superimposed monomeric secondary structure of ClpK (red) and ClpB (1QVR-B) template (blue). (**C**) Secondary structure elements of ClpK domains colored as follows: Blue; N-terminal domain, red; D1 large domain, green; D1 small domain, grey; short linker region, pink; D2 large domain, light blue; D2 small domain/C-terminal domain. The structures were refined using OPLS_2005 force field and visualized using PYMOL [35,36].

**Figure 7:**
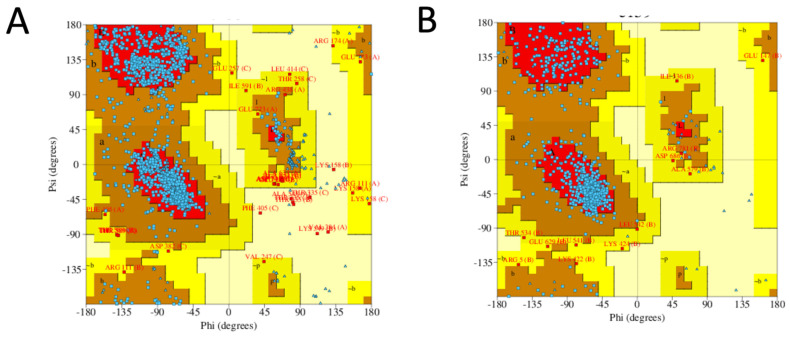
Ramachandran plots of the modelled ClpK protein and the template ClpB protein. (**A**) ClpK trimeric protein. (**B**) ClpB template protein. The blue dots represent the residues of each protein. The regions are denoted as follows: the most favoured regions (red), additional allowed regions (brown), the generously allowed regions (yellow) and the disallowed regions (light yellow). The figures were obtained from the ProCheck server.

The modelled ClpK structure was validated using the ProCheck server and is shown in Figure 6A. ATPases can exist in a monomeric, dimeric and trimeric state in the absence of nucleotides, therefore the hypothesized trimeric structure of ClpK is not alarming [23]. Ramachandran analysis of the trimeric ClpK (90.10%) and template ClpB (83.10%) proteins showed that the majority of their protein residues lie within the most favoured regions (Figure 7, Table S1). A Rama Z-score of –0.75 ± 0.16 was obtained for the trimeric ClpK structure from the MolProbity server, this value was within the accepted Z-score range. The structural alignment between the monomeric ClpK and ClpB (Figure 6B) gave a root mean square deviation (RMSD) value of 0.300 Å which is indicative of the two structures adopting a similar conformation [33]. The modelled structure of ClpK is consistent with other known structures of ATPases which contain a mixture of α-helices and β-sheets (Figure 5 and Figure 6). Furthermore, this structure agrees with the virtual CD data obtained for ClpK using the DichroCalc server and DicroWeb analysis (Figure S1, Table S2). The spectrum shows that ClpK displays one ellipticity maxima at about 190 nm, and one ellipticity minima at about 220 nm which is characteristic of proteins consisting mainly of α-helices [34].

The comparison of ClpK N-terminal domain with other Clp ATPases shows that it contains a 100 amino acid N-terminal extension which is unique to this protein [9]. The role of this N-terminus extension is not known however its presence suggests a possible unique role of the ClpK N-terminus in protein homeostasis. The ClpB N-terminal domain contains a substrate binding groove which is known to recognise hydrophobic residues of unfolded or aggregated proteins [37]. A similar substrate groove was identified in the ClpK N-terminal domain using the DoGSiteScorer (Figure S2, Table S3). Therefore, it could be hypothesized that the ClpK binding pocket recognizes substrates in a similar manner to ClpB.

The NBD1 and NBD2 domain of ClpK adopts a RecA-like fold characterized by a central β-sheet flanked by α-helices (Figure S3). This fold is a common structural feature found in most ATPases and assists with the movement of polypeptides into the proteolytic core, which is a critical step in protein proteolysis [21,38]. In most Clp proteins, NBD1 and NBD2 are not separated; however, in ClpK we find that NBD1 and NBD2 are separated by a short linker sequence which adopts a helical structure (Figure 5 and Figure 6B). In ClpB, this linker region is termed “the middle domain” and is essential for chaperone activity, although its exact function has not been fully established [21]. We have observed that the linker region of ClpK and ClpB differ in amino acid length, with the linker region of ClpB being almost double the size of ClpK. The role of the linker region in ClpK is yet to be established. One could envisage that it may transport a different range of substrates compared to those transported by ClpB. 

### 2.4. Molecular Dynamics Simulation

**Figure 8:**
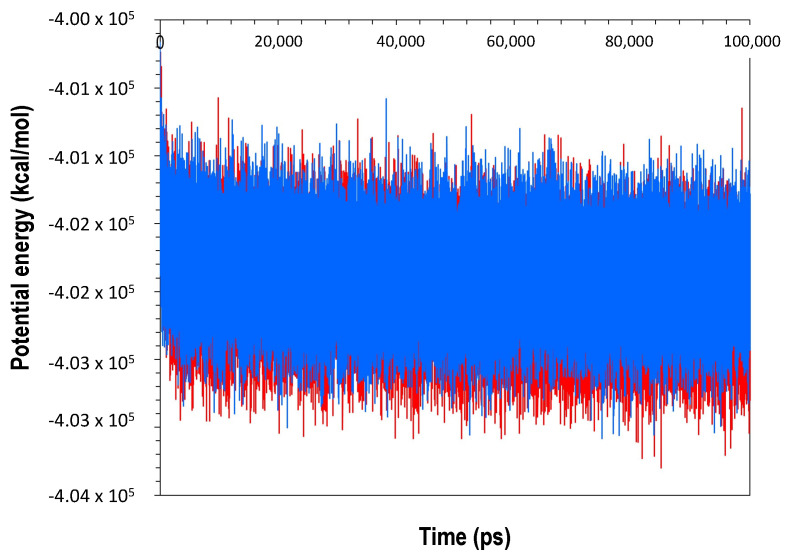
Potential energy profile of ClpK and ClpB during 100,000 ps molecular dynamic simulation. The potential energy for ClpK and ClpB is shown in red and blue, respectively. The image was generated using GraphPad prism.

To explore the dynamic behavior and stability of the modelled structure, molecular dynamics (MD) simulations and post-dynamic analyses were carried out. ClpB was used as a control for all the MD simulations and post-dynamic analyses. Figure 8 shows the potential energy profiles to compare the trajectory of the alpha carbons (Cα) within a time frame for ClpK and ClpB. A comparison of the potential energy obtained for ClpK (−401,975.3 ± 313.1 kcal/mol) and ClpB (−468,132.8 ± 331.6 kcal/mol) shows a slight, insignificant shift, thus indicating that the ClpK structure was adequately modelled.

**Figure 9:**
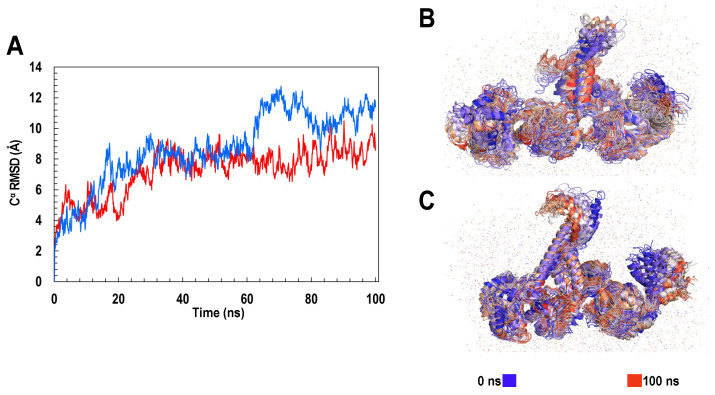
Trajectory analysis showing the RMSD values and the conformational changes of ClpK and ClpB over 100 ns. (**A**) RMSD values of ClpK and ClpB over 100 ns. The trajectory of ClpK is indicated in red and the trajectory of ClpB is indicated in blue. (**B**) Conformational changes of ClpB observed over 100 ns. (**C**) Conformational changes of ClpK observed over 100 ns.

**Figure 10:**
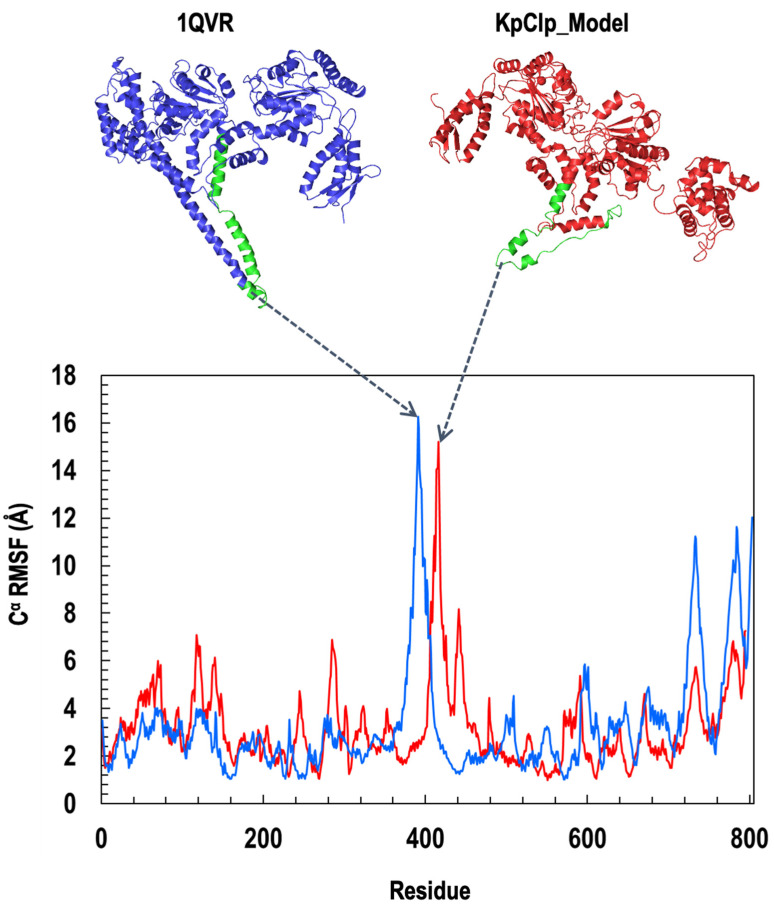
The RMSF of the ClpK and ClpB residues as a function of the 800 ns simulation time. ClpK is represented as red and ClpB is represented as blue on the graph and structures. The positions of the peaks are represented on the ClpK and ClpB structures in green. The highest peak for ClpK is seen around residues 410 to 425, while the highest peak for ClpB is seen around residues 379 to 407. To assess the regions of flexibility, we add 110 and 4 to the region values obtained for ClpK and ClpB, respectively, as the structures have been modelled from residue 110 and 4. The graph was generated using Excel. The 3D protein structures were generated using PyMOL.

To assess the dynamic nature of ClpK we calculated the root mean squared deviation (RMSD) and the root mean square fluctuation (RMSF) values. Figure 9A shows the RMSD values of ClpK (7.22 ± 1.52 Å), which increases from 2 Å to 9 Å over 100 ns. A similar increase in RMSD values is observed for ClpB (8.68 ± 2.37 Å), which increases from 2 Å to 11 Å over 100 ns. The increasing RMSD values observed indicate significant conformational changes, which are shown in Figure 9B,C for ClpB and ClpK, respectively. The conformational changes observed over 100 ns are consistent with the dynamic nature of proteins [39,40]. Furthermore, we used RMSF values to identify amino acids in a protein which contribute the most to protein flexibility: the higher the RMSF value the greater the flexibility [34]. The D1 small domain and linker region were identified as regions which contribute to the flexibility of ClpK (3.17 ± 1.73 Å) and ClpB (3.27 ± 2.27 Å) (Figure 5 and Figure 10). The role of the ClpK and ClpB linker regions would have to further investigated to assess whether the motion of the amino acids plays a role in protein function and stability.

**Figure 11:**
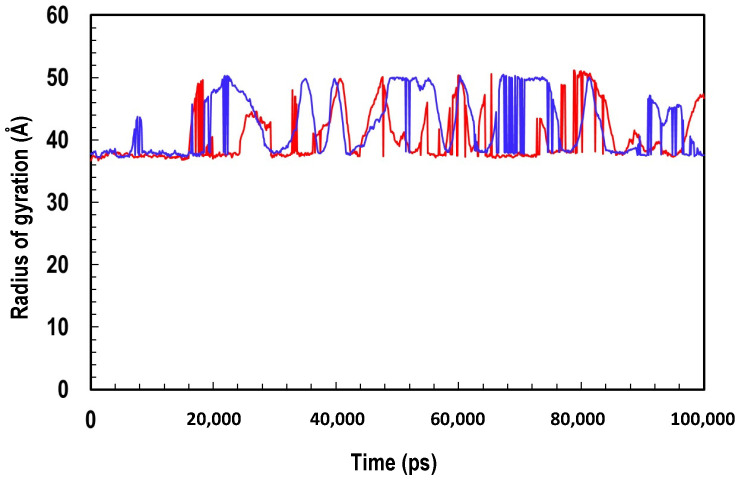
Trajectory analysis showing the Radius of gyration of the Cα of ClpK over 100,000 ps. ClpK is represented as red, and ClpB is represented as blue.

The radius of gyration (Rg) represents the compactness of a structure [41]. The values obtained for ClpK (40.4 ± 3.93 Å) and ClpB (42.37 ± 4.71) indicate that the proteins do not differ much in terms of structure compactness (Figure 11). Additionally, the features of the gyration profiles of ClpK and ClpB indicate structural transformation, suggesting that the proteins are constantly transforming during simulation [42]. ClpB seems to undergo transformational change at around 10,000 ps, while ClpK only undergoes transformation around 20,000 ps (Figure 11). The variation in the Rg profiles across the simulation time once again indicates that both these proteins are structurally dynamic (Figure 9 and Figure 11).

### 2.5. Protein Disorder Prediction

Following homology modelling and MD simulation, we assessed the structure of ClpK for protein disorders and binding disorders. This allowed us to determine if it was possible to express and purify soluble ClpK as an initial step to protein characterization and protein-drug interaction studies. Disordered protein regions do not adopt a stable confirmation and therefore make protein purification, protein-ligand binding studies and crystallization difficult [43]. 

**Figure 12:**
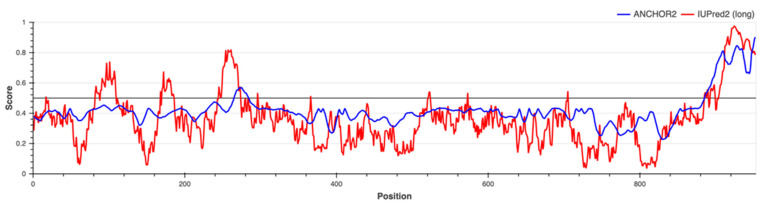
Protein disorder prediction for ClpK. The ClpK protein sequence (Figure S4) was analysed using the IUPred2A server for the presence of ordered and/or disordered regions. The black line represents the threshold; the red line represents the protein disorder prediction (IUPred2A), and the blue line represents the binding disorder prediction (Anchor2).

Figure 12 shows that less than 40% of the ClpK residues were predicted to be disordered through IUPred2A (red line), suggesting that ClpK can be expressed and purified [43]. IUPred2A predicts some of the disordered protein residues to be situated in the N-terminal domain (3 small peaks), while most of the disordered protein residues are seen in the C-terminal domain (Figure 12). It has been noted, that proteins with disordered regions carry out important functional roles such as phosphorylation, regulation and protein-DNA binding [43]. Using the Anchor2 server, we observed that a majority of the disordered binding regions were situated in the C-terminal domain (Figure 12). Further studies could focus on investigating molecules that binds to the C-terminal domain to facilitate the transition from a disordered to ordered state.

### 2.6. Expression and Purification of ClpK 

**Figure 13:**
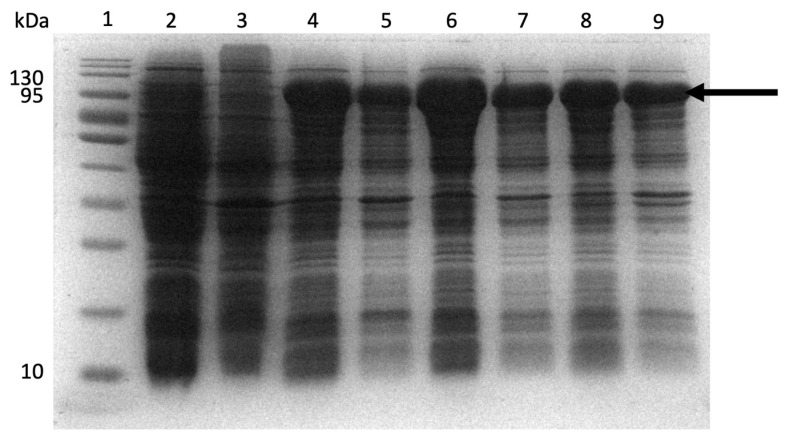
ClpK expression using varying IPTG concentrations. Protein expression was induced with varying IPTG concentrations (0.1 mM, 0.25 mM and 0.5 mM) at 15 °C for 24 h. Following expression, the culture was harvested by centrifugation. The resulting pellet was resuspended in lysis buffer, sonicated and analysed for soluble and non-soluble protein on a SDS-PAGE gel. Lane 1: molecular weight marker; Lane 2 and Lane 3: uninduced supernatant (Sn) and uninduced pellet, respectively. Lane 4: 0.1 mM Sn; Lane 5: 0.1 mM pellet; Lane 6: 0.25 mM Sn; Lane 7: 0.25 mM pellet; Lane 8: 0.5 mM Sn; Lane 9: 0.5 mM pellet. The arrow indicates the position of the expected protein.

To our knowledge, the expression and purification of ClpK has not been reported to date. To test the expression of ClpK, *E. coli* BL21 cells were transformed with pCold-I plasmid containing the *clpk* gene (ClpK construct). Different expression conditions were tested to determine suitable conditions to express the soluble ClpK protein. Figure 13 shows the successful induction and expression of soluble ClpK using 0.1 mM, 0.25 mM, and 0.5 mM Isopropyl β-D-1-thiogalactopyranoside (IPTG) respectively, as indicated by the protein band corresponding to the molecular weight of ClpK (Figure 13). Based on the band intensity of expressed ClpK, 0.25 mM IPTG was selected as an optimal concentration for expression (Figure 8, Lane 6).

**Figure 14:**
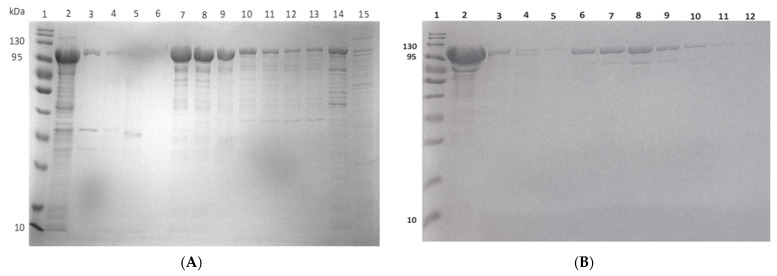
Purification of ClpK using anion exchange and affinity chromatography. (**A**) Anion exchange purification. Lane 1: molecular weight marker; Lane 2: crude sample; Lane 3: flow through; Lane 4: unbound (wash with buffer A); Lane 5: wash with 20% Buffer B; Lane 6–14: elution with 40% Buffer B; Lane 15: elution with 100% Buffer B. Elution samples were collected in 1 mL fractions. (**B**) Affinity purification. The eluents from the anion exchange purification were used for further purification. Lane 1: molecular weight marker; Lane 2: load; Lane 3: flow through; Lane 4: unbound wash; Lane 5–11: elution with buffer C; Lane 12: elution with Buffer D.

Following expression, ClpK was purified using ion exchange and affinity chromatography. Initially, the expressed protein was subjected to ion exchange chromatography, which is based on the electrostatic interaction between the resin and the protein [44]. At pH 7.4, ClpK (pI: 5.61) is negatively charged; therefore it binds to the positively charged anion exchange resin. The protein bound to the resin was eluted with increasing concentrations of sodium chloride in Buffer B (Figure 14A). ClpK co-elutes with impurities even at the highest salt concentration: therefore, a second purification step was performed. In addition, the recombinant protein only reached 2.7% purity after ion exchange chromatography (Table 3). The eluent from anion exchange was passed through an affinity chromatography column. ClpK was expected to bind to the affinity column resin since it contains a HisTag, while the contaminating proteins were expected to flow through [44]. As shown in Figure 14B and Table 3, the partially purified ClpK was successfully purified to homogeneity (Figure 14B, Lane 6 to 10). The specific activity increased from 0.0423 units/mg in the supernatant to 11.13 units/mg in the homogenous protein sample (Table 3). The specific activity is of importance, as it can be used to determine the purity of a protein following a dual purification procedure [45]. 

### 2.7. Biophysical Characterisation of ClpK

#### 2.7.1. Enzyme Activity Assay

**Figure 15:**
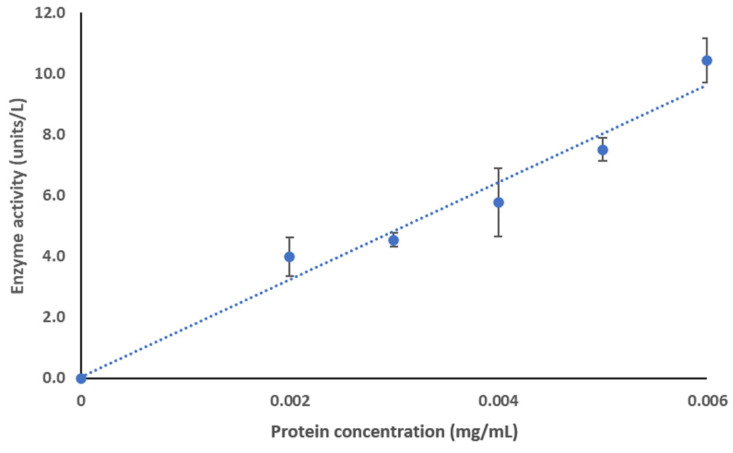
ClpK ATPase activity. ATPase activity was determined by incubating protein at varying concentrations (0–0.006 mg/mL) in reaction buffer containing 40 mM Tris, 80 mM NaCl, 8 mM MgAc^2^, 1 mM EDTA, and 4 mM ATP (pH 7.5) for 30 min. The release of phosphate ions was determined at 620 nm. The absorbance values were then used to calculate enzyme activity. Each data point is an average of three independent measurements, and the error bars represent standard deviation. Equation: y = 1600.4x + 0.0361, R^2^: 0.9677.

ClpK has been identified as an ATPase since it consists of structural motifs associated with the hydrolysis of ATP [14,16,17,19]. To our knowledge, the ability of ClpK to hydrolyse ATP in vitro has not been reported to date. Therefore, we tested the biological activity of the purified protein using an ATPase assay. Figure 15 shows the ATPase activity of ClpK. Enzyme activity increases from 0 ± 0 units/L to 10.43 ± 0.72 units/L as protein concentration increases from 0 mg/mL to 0.006 mg/mL, therefore indicating that ATP hydrolysis is concentration dependent (Figure 15).

#### 2.7.2. Far UV-CD Spectroscopy

**Figure 16:**
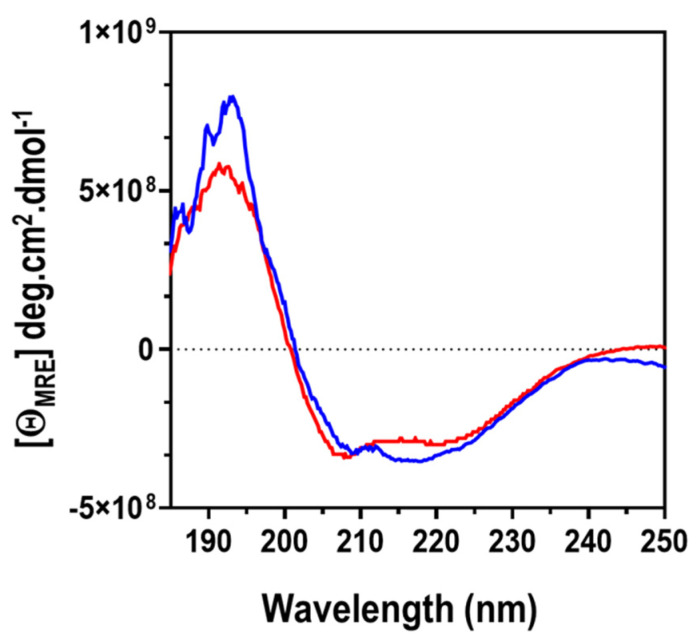
Far-UV CD spectroscopy of ClpK in the presence and absence of ATP. CD spectra of ClpK (2 µM) at pH 7.4 in the presence of 0.2 mM ATP (blue spectrum) and in the absence of ATP (red spectrum). Each spectrum represents an average of five accumulated spectra derived at 20 °C using a 2 mm path length quartz cuvette.

Assessing the CD data of a protein over the far-UV range (185–250 nm) produces a CD spectrum which is indicative of the fingerprint of the secondary structure of the protein or peptide [47]. Therefore, we used CD spectroscopy to explore the secondary structure of ClpK in the presence and absence of ATP. The CD spectra in the presence and absence of ATP displayed a high α-helical and lower β-structural content (Figure 16). The CD spectra for ClpK shows a negative minima at 208 ± 1 and 220 ± 1 nm and a maxima at 192 ± 1 nm in the absence of ATP. The CD spectra obtained from practical analysis correlates with the structure used in modelling ClpK (Figure 6 and Figure S1). We observed that in the presence of ATP, the maxima increased to 193 ± 1 nm. There seems to be a slight modification in the secondary structure content upon ATP binding, which is not unusual because ClpK contains two ATP binding sites and is considered to be an ATPase.

#### 2.7.3. Extrinsic Fluorescence Spectroscopy

**Figure 17:**
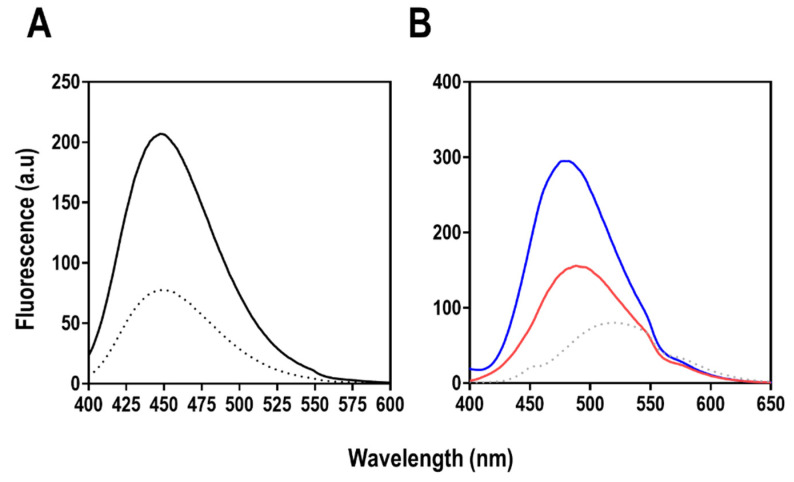
Tertiary structure analysis of ClpK using extrinsic fluoresence spectroscopy. (**A**) Fluorescence emission spectra of mant-ATP in the presence (bold spectrum) or absence (dotted line spectrum) of 2.0 µM ClpK. The fluorescent nucleotides (10 µM) were excited at 355 nm and emissions were collected between 400 and 600 nm. Each spectrum represents an average of five accumulated spectra from three independent experiments. (**B**) ANS fluorescence spectroscopy of ClpK. ClpK (2.0 µM) was incubated (20 °C, 30 min) with ANS alone (red spectrum) and ANS in the presence of 0.2 mM ATP (blue spectrum). The dotted line spectrum represents spectrum of free (unbound) ANS in the assay buffer (10 mM sodium phosphate pH 7.4 and 5 mM MgCl_2_). The fluorescence emission was recorded in the wavelength region 400–650 nm after exciting the samples at 390 nm. The bandwidths were set at 5 nm for excitation and emission. The path length of the sample was 1 cm. Each spectrum represents an average of five accumulated spectra from three independent experiments. Here, a.u is arbitrary unit.

Tertiary structure analysis was carried out using extrinsic fluorescence spectroscopy based on mant-ATP and ANS fluorescence (Figure 17). Both mant- and ANS are fluorophores that alter their spectral properties depending on the polarity of their environment [48,49]. When mant and ANS bind within a hydrophobic pocket in a protein, an increase in quantum yield with a concomitant maximum emission wavelength (λ_max_) is observed [48,49]. This degree of shift in λ_max_ often depends on the degree of hydrophobicity of the environment [48,49].

Mant-ATP fluorescence was used to further establish the presence of an ATP binding site (Figure 17). The spectrum indicates binding of mant-ATP as indicated by an increase in the fluorescence quantum yield at 450 nm compared with free mant-ATP. However, there was no shift observed upon the binding of mant-ATP (blue region of the spectrum) at about 450 nm. This indicates that the local environment of mant-ATP interaction may not be hydrophobic. This is expected because ATP is highly hydrophilic and will require hydrophilic amino acid side chains and Mg^2+^ for interaction. Beyond just interacting with ATP, this piece of information further confirms that the recombinant protein shows activity towards ATP. Furthermore, an ANS binding assay was used to observe the presence of hydrophobic patches on ClpK in the presence or absence of ATP. ClpK, in the presence and absence of ATP showed a substantial increase in quantum yield when excited at 390 nm in the presence of ANS when compared to free ANS. There was also a shift in maximum emission wavelength from 520 nm (free ANS) to 481 nm and 489 nm for ClpK:ATP, and only ClpK, respectively. This is indicative of ANS accessing hydrophobic pockets in ClpK.

In summary, we used bioinformatic analysis to investigate the distribution of Clp proteins, particularly ClpK, across seven *Klebsiella* species. Our data showed that the distribution of *clpk* was considerably less compared to that of *clpa*, *clpb* and *clpx*. Additionally, a distinguishing structural feature of ClpK amongst the Class I proteins is a shorter middle domain linking NBD1 and NBD2. With this domain implicated in recognising substrates for unfoldase activity, ClpK could potentially recognise a varying substrate range. Therefore, future studies should investigate the role of the ClpK middle domain. 

This study is the first to report the conditions for the expression and purification of ClpK. Furthermore, we characterised the biophysical properties ClpK and demonstrated that purified ClpK is biologically active. This gives researchers an opportunity to work towards developing mechanisms to target the thermal stability of *K. pneumonia* as an alternative approach to antibiotic therapy.

## 3. Materials and Methods

### 3.1. Species and Databases

The protein genomes of 100 *Klebsiella* strains (7 species, complete draft) were collected from the National Center for Biotechnology Information (NCBI) Genome database. The 100 strains which were analyzed included: 8 *K. aerogenes* strains, 1 *K. michiganensis* strain, 7 *K. oxytoca* strains, 57 *K. pneumoniae* strains, 15 *K. pneumonia* subsp. *pneumoniae* strains, 3 *K. quasipneumoniae* strains, and 9 *K. variicola* strains. The various *Klebsiella* species, strains, web-links, and references are presented in the supplementary database (Table S3).

### 3.2. Genome Data Mining and Annotation of Caseinolytic Proteins

Clp proteins were mined from different *Klebsiella* species by firstly, obtaining the protein file of each species. Each file was then individually searched for the presence of Clp proteins. The sequence for each Clp protein was separated from the main file and used for further analysis.

### 3.3. Phylogenetic Analysis of Clp Proteins

Phylogenetic analysis of the Clp proteins was carried out using the method described by Ngcobo, et al. (2020) [50]. Briefly, the protein sequences were aligned using MAFFT v6.864 embedded on the Trex Web Server [51,52]. The alignments were automatically deduced and optimized by the Trex Web server. The file for the best tree was then visualized and colored using the Interactive Tree of Life (iTOL) server [53].

### 3.4. Clp Protein Homology Analysis

The percentage identity between different protein codes within the Clp classes were analyzed using Clustal Omega [54]. Full length Clp protein sequences were subjected to Clustal analysis to obtain the percentage identity amongst the proteins as identity matrix results. The results were then laid out on an Excel spreadsheet for analysis. 

The percentage identity between the different Clp classes (ClpA, ClpB, ClpK and ClpX) was analyzed in the above-mentioned way.

### 3.5. Homology Modelling of ClpK

To model the structure of ClpK (Uniprot: E0W6V3), a template search was done on PYHRE [55], Itasser [56,57,58] and NCBI [59]. The template was selected based on high sequence identity, coverage parameters and a high determination resolution. Thus, *Thermus thermophilus* ClpB (1QVR-B) was selected sharing 52% identity and 83% query coverage with the target protein. The modelled ClpK and template ClpB was submitted to ProCheck for stereochemical analysis using Ramachandran analysis [60]. The MolProbity server was used to obtain the Rama Z-score for the trimeric ClpK structure [61]. The model was then submitted to the Maestro v12.2 molecular modelling algorithm [62]. To pre-process the protein structure, bond orders were assigned, hydrogen atoms were added, zero bond orders to metals and disulphide bonds were created, and water molecules that were 5 Å from heteroatoms were deleted. Additionally, the PROPKA algorithm was used at pH 7.0 to optimize the hydrogen bonding network by sampling the orientation of water molecules. Lastly, the OPLS_2005 force field was used to refine the structure through minimisation. The stereochemistry of the side chains was checked to ensure that no major perturbations have been induced while preparing the structure. The minimized structure was saved as Maestro (.mae) file for subsequent prediction analysis.

The ClpK and ClpB sequences were aligned on TCOFFEE to prepare for modelling [32]. The position of Walker A and Walker B motifs on the template protein was assigned as shown in Lee, et al. (2003) [21]. The final ClpK structure was then visualized using PyMol, and the RMSD value was obtained [54]. The monomeric ClpK and template ClpB structures were processed through the Protein*Plus* (https://proteins.plus, accessed on 15 July 2021) to identify the position of binding pockets in each protein structure [63].

### 3.6. Molecular Dynamics Simulation

Molecular dynamics (MD) simulations were carried out on Maestro v12.2 using the implemented GPU-enabled Desmond molecular dynamics simulation engine. The ClpK trimeric protein or the template ClpB protein were saved as PDB files and submitted to the Linux (Ubuntu) desktop server for the Desmond MD simulations studies. The ClpK trimeric protein or ClpB template protein were placed in an orthorhombic box (distance from the box face to the outermost protein atom was set 10 Å, and the box angle was α = β = γ = 90°). The volume box containing the ClpK trimeric protein or ClpB template protein was minimized, and counter ions were added to neutralize the system. A total of 0.15 M NaCl was added into the solvent box for physiological conditioning. After solvation and ionization, the system was submitted for the MD simulation. MD simulation is divided into eight distinct stages in which the simulation parameters are specified for each stage. Stages 1–7 are the equilibration, which is made up of short simulation steps, and stage 8 is the final long range 100 ns simulation stage. The type and parameters of the solvated system were detected in stage 1. In stage 2, a 100 ps simulation was carried out using Brownian dynamics under NVT conditions at 10 K with restraints placed on the solute-heavy atoms. Stage 3 involved a 12 ps simulation under NVT conditions at 10 K with restraints on heavy atoms. Stages 4, 6 and 7 (the pocket solvation at stage 5 was skipped) employed short simulation steps (12, 12 and 24 ps, respectively) under NPT conditions (at 10 K and with restraints on heavy atoms for stages 4 and 6). No restraints were placed on heavy atoms at stage 7. The final MD production stage at a constant temperature of 300 K was carried out at stage 8.

### 3.7. Post-Dynamic Analysis

Post dynamic analyses of the trajectories derived from the MD simulation studies were carried out using Schrodinger Maestro v12.2 or the Bio3D R statistical package for comparative analysis of protein structures. Firstly; Simulation Quality Analysis (implemented in Maestro v12.2) was used to analyse the quality of simulations which analyse the average energy, pressure, temperature and volume. Secondly, the Simulation Interaction Diagram algorithm (implemented in Maestro v12.2) was used to analyse the root- mean-square-deviation (RMSD) of the alpha carbon atoms (Cα), the root-mean- square fluctuations (RMSF) of the residues, and to carry out a secondary structure element analysis. Lastly, the Simulation Events Analysis algorithm (implemented in Maestro v12.2) was used to calculate the radius of gyration (Rg) and atomic distance.

### 3.8. Protein Disorder and Circular Dichroism Analysis

The primary amino acid sequence of ClpK was used to predict protein disorder and perform a virtual circular dichroism (CD). The sequence was subjected to analysis on the IUPred2A server for protein disorder prediction [64]. The sequence was also subjected to analysis on DichroCalc which is found on The Hirst Group Home Page (https://comp.chem.nottingham.ac.uk/dichrocalc/, accessed on 28 May 2020) server. The CD graph obtained from DichroCalc was then analyzed for secondary structures both manually and through the DicroWeb server [65].

### 3.9. ClpK Expression and Purification

The cDNA encoding for ClpK (Uniprot: E0W6V3) was extracted from GenBank. The gene was synthesized, codon-optimized and cloned into a pColdI vector using BamH1 and SalH1 by GenScript, Piscataway, New Jersey, United States. The resulting plasmid (pCold1-ClpK) was transformed into *Escherichia coli* BL21 cells. The *E. coli* BL21 pColdI-ClpK glycerol stocks (100 μL) were used to inoculate 10 mL lysogeny broth (LB) containing 100 mg/mL ampicillin (10 μL). The cells were grown overnight (16 h) at 37 °C. Cells from the overnight flask (1 mL) were then used to inoculate flasks containing 100 mL LB agar and 100 mg/mL ampicillin (100 μL). The cells were grown to an OD of 0.4 to 0.6 before they were cold-shocked in ice for 30 min. The culture was then induced with 0.25 mM isopropyl-β-D-thiogalactopyranoside (IPTG) and incubated at 15 °C for 24 h. Uninduced cells served as the control and were grown along with the induced cells.

Both uninduced and induced cells were harvested by centrifugation at 6800× *g* for 15 min. After centrifugation, the resulting pellets were resuspended in binding buffer (20 mM sodium phosphate, 20 mM imidazole, pH 7.4). The cells were then ruptured by sonication (15 min, 30 s on, 20 s off, 50% amplification) followed by centrifugation. Both the soluble and insoluble fractions were analyzed using reducing 12.5% SDS-PAGE [66]. The protein concentration was determined using a Thermofisher NanoDrop 2000 at 280 nm [67].

Purification was carried out using firstly; a 5 mL HiTrap Q HP anion exchange column (GE Healthcare, Chicago, IL, USA), and secondly; a 1 mL HisTrap HP column (GE Healthcare, Chicago, IL, USA). The HiTrap Q HP column was equilibrated with binding buffer. The soluble cell fraction was passed through the anion exchange column. Unbound proteins were removed from the column using binding buffer, and any remaining protein was eluted with Buffer B (20 mM imidazole, 20 mM sodium phosphate, and 0.2 M NaCl, pH 7.4).

Fractions containing the eluted protein were pooled and passed through the HisTrap HP column which had been equilibrated with Buffer B (20 mM imidazole, 20 mM sodium phosphate, and 0.2 M NaCl, pH 7.4). Unbound proteins were removed from the column using Buffer B, and the remaining proteins was eluted with Buffer C (192 mM imidazole, 20 mM sodium phosphate, and 0.5 M NaCl, pH 7.4), the elution samples were collected and analyzed using reducing 12.5% SDS-PAGE gels [66].

### 3.10. Enzyme Activity Assay

ATP hydrolysis was measured using the ATPase/GTPase Activity Assay Kit (Lot# 113BI08A16) from Sigma-Aldrich according to the manufacturers’ instructions. Briefly; 0 to 0.006 mg/mL ClpK was mixed with 30 μL of reaction buffer (40 mM Tris, 80 mM NaCl, 8 mM MgAc^2^, 1 mM EDTA, and 4 mM ATP, pH 7.5) and incubated at room temperature for 30 min. The ATPase activity was followed by measuring the release of phosphate ions spectrometrically at 620 nm, as a result of the conversion of ATP to ADP. Measurements were performed in triplicate.

### 3.11. Far-UV Circular Dichroism Spectroscopy

The secondary structure contents of the protein were analysed with far-UV circular dichroism using 2 µM of ClpK in 5 mM sodium phosphate pH 7.4. This was performed both in the absence and presence of 0.2 mM ATP. The Jasco J-1500 spectropolarimeter was used to conduct the experiment at 20 °C using a 2 mm quartz cuvette. Spectra measurements were collected in 5 accumulations from 250 to 180 nm at 2.5 nm band width, 0.2 nm data pitch and 1 s response time. The CD spectra was recorded in millidegrees ellipticity (θ_mdeg_) and was later converted to mean residue ellipticity [θ_MRE_] (deg·cm^2^·dmol^−1^). This was calculated using the equation below:

(1) $$[\theta_{\mathrm{MRE}}]=\frac{100\times \theta_{\mathrm{mdeg}}}{\mathrm{cnl}}$$

where θ_mdeg_ is the signal of measured ellipticity in mdeg, c is the protein concentration measured in mM, n is the number amino acid residues of the protein, and l is the pathlength in cm. The data were analysed and processed using the Dichroweb algorithm by employing the CONTIN parameter.

### 3.12. Extrinsic Fluorescence Spectroscopy

The ATP substrate binding to ClpK was probed using methylanthraniloyl-ATP (mant-ATP). A concentration of 10 µM mant-ATP each was added to the protein and was excited at 355 nm while the excitation and emission band widths were fixed at 2.5 nm. The emission was collected between 400–600 nm wavelengths in three accumulations.

Extrinsic ANS fluorescence was performed to probe for hydrophobic pockets on the protein. A concentration of 200 µM ANS was incubated with the protein for 1 h in the dark, and the samples were excited at 390 nm wavelength. Emission spectra were collected between 400 and −600 nm wavelengths at 5 nm excitation and emission bandwidths using 200 nm/min scanning speed. The experiments were performed using Jasco FP-6300 fluorescence spectrophotometer at 20 °C using a 10 mm quartz cuvette.

All fluorescence samples were prepared with 2 µM protein concentration in the presence and absence of 2 mM ATP using 10 mM sodium phosphate pH 7.4 and 5 mM MgCl2 for ATP-bound samples. Buffer contributions were subtracted from each final spectrum.

## Figures and Tables

**Table 1:** Clp regulatory subunits identified across different proteins, exhibiting diverse functions.

**Class I**			
**Clp Regulatory Subunits**	**Species**	**Functions**	**References**
ClpA ^1^	Gram-positive Proteobacteria	Protein quality control	[20]
ClpB	Prokaryotes, yeast, and plants	Disaggregation of stress-damaged proteins	[21,22,23]
ClpC	Gram-positive bacteria (Firmicutes and Actinobacteria) and Cyanobacteria	Protein quality control	[20]
ClpD	Chloroplasts of higher plants	Molecular chaperone	[23]
ClpE	Firmicutes	Thermotolerance, cell division and virulence	[20]
ClpK	*K. pneumonia*	Thermotolerance	[13]
ClpL	*Streptococcuss pneumoniae*	Nucleotide phosphohydrolase activity, stabilises unfolded proteins, prevents protein aggregation	[11]
ClpV	Gram-negative bacteria	Component of the type V1 secretion system	[24]
**Class II**			
**Clp Regulatory Subunits**	**Species**	**Functions**	**References**
ClpM	*Mus musculus*	Protein quality control	[23,25]
ClpN	*Pseudomonas aeruginosa*	Cell division	[23,25]
ClpX	Proteobacteria, Firmicutes and Thermatogae	Protein quality control, cell division and virulence	[20]
ClpY	Gram-positive Proteobacteria	Cell division, heat shock response, and capsule transcription	[20]

^1^ ClpA and ClpC are orthologs; bacteria usually contain either one of these [20].

**Table 2:** Percentage identity of the various Clp proteins obtained through Clustal analysis.

Clp Protein	Identity (%)
ClpA	97.1–100
ClpB	96.5–100
ClpX	98.3–100
ClpK	94.1–100

**Table 3:** Purification table for ClpK.

Steps	Volume (ml)	Protein (mg/mL) ^b^	Total Protein (mg)	Specific Activity (units/mg) ^c^	Total Activity (units)	Yield (%)	Purity (%)
Crude extract ^a^	30	0.0345	93.10	0.0423	3.94	100	0.38
Anion eluant ^d^	30	0.0122	32.94	0.302	9.95	35.4	2.7
HisTag eluant ^e^	10	0.117	1.17	11.13	13.02	1.26	100

^a^ Soluble fraction obtained from 0.77 g of wet weight *E. coli* cell pellet (from 1 L of bacterial culture); ^b^ Protein concentration determined by Bradford assay using BSA as a standard protein [46]. ^c^ Calculated using the ATPase assay; the release of phosphate ions is measures as ATP is converted to ADP (A_620nm_). ^d^ Elution collected from the ion exchange column. ^e^ Pooled eluant collected from the HisTag column.

## Supplementary Materials

The following are available online.

## References

[1] Santajit S., Indrawattana N. (2016). Mechanisms of Antimicrobial Resistance in ESKAPE Pathogens. BioMed Res. Int..

[2] Martin R.M., Bachman M.A. (2018). Colonization, infection, and the accessory genome of *Klebsiella pneumoniae*. Front. Cell. Infect. Microbiol..

[3] Pendleton J.N., Gorman S.P., Gilmore B.F. (2013). Clinical relevance of the ESKAPE pathogens. Expert Rev. Anti-Infect. Ther..

[4] Brisse S., Passet V., Grimont P.A. (2014). Description of *Klebsiella quasipneumoniae* sp. nov., isolated from human infections, with two subspecies, *Klebsiella quasipneumoniae* subsp. *quasipneumoniae* subsp. nov. and *Klebsiella quasipneumoniae* subsp. *similipneumoniae* subsp. nov., and demonstration that *Klebsiella singaporensis* is a junior heterotypic synonym of *Klebsiella variicola*. Int. J. Syst. Evol. Microbiol..

[5] Chapman P., Forde B.M., Roberts L.W., Bergh H., Vesey D., Jennison A.V., Moss S., Paterson D.L., Beatson S.A., Harris P.N. (2020). Genomic investigation reveals contaminated detergent as the source of an ESBL-producing *Klebsiella michiganensis* outbreak in a neonatal unit. J. Clin. Microbiol..

[6] Doorduijn D.J., Rooijakkers S.H., van Schaik W., Bardoel B.W. (2016). Complement resistance mechanisms of *Klebsiella pneumoniae*. Immunobiology.

[7] Joainig M.M., Gorkiewicz G., Leitner E., Weberhofer P., Zollner-Schwetz I., Lippe I., Feierl G., Krause R., Hinterleitner T., Zechner E.L. (2010). Cytotoxic effects of *Klebsiella oxytoca* strains isolated from patients with antibiotic-associated hemorrhagic colitis or other diseases caused by infections and from healthy subjects. J. Clin. Microbiol..

[8] Singh L., Cariappa M., Kaur M. (2016). *Klebsiella oxytoca*: An emerging pathogen?. Med. J. Armed Forces India.

[9] Bojer M.S., Struve C., Ingmer H., Hansen D.S., Krogfelt K.A. (2010). Heat resistance mediated by a new plasmid encoded Clp ATPase, ClpK, as a possible novel mechanism for nosocomial persistence of *Klebsiella pneumoniae*. PLoS ONE.

[10] AhYoung A.P., Koehl A., Cascio D., Egea P.F. (2015). Structural mapping of the C lp B ATP ases of *Plasmodium falciparum*: Targeting protein folding and secretion for antimalarial drug design. Protein Sci..

[11] Park S.S., Kwon H.Y., Tran T.D., Choi M.H., Jung S.H., Lee S., Briles D.E., Rhee D.K. (2015). ClpL is a chaperone without auxiliary factors. FEBS J..

[12] Zolkiewski M., Zhang T., Nagy M. (2012). Aggregate reactivation mediated by the Hsp100 chaperones. Arch. Biochem. Biophys..

[13] Bojer M.S., Struve C., Ingmer H., Krogfelt K.A. (2013). ClpP-dependent and-independent activities encoded by the polycistronic clpK-encoding locus contribute to heat shock survival in *Klebsiella pneumoniae*. Res. Microbiol..

[14] Maurizi M.R., Xia D. (2004). Protein binding and disruption by Clp/Hsp100 chaperones. Structure.

[15] Miller J.M., Chaudhary H., Marsee J.D. (2018). Phylogenetic analysis predicts structural divergence for proteobacterial ClpC proteins. J. Struct. Biol..

[16] Schirmer E.C., Glover J.R., Singer M.A., Lindquist S. (1996). HSP100/Clp proteins: A common mechanism explains diverse functions. Trends Biochem. Sci..

[17] Kress W., Maglica Ž., Weber-Ban E. (2009). Clp chaperone–proteases: Structure and function. Res. Microbiol..

[18] Filloux A., Hachani A., Bleves S. (2008). The bacterial type VI secretion machine: Yet another player for protein transport across membranes. Microbiology.

[19] Mfeka M.S., Martínez-Oyanedel J., Chen W., Achilonu I., Syed K., Khoza T. (2020). Comparative analyses and structural insights of new class glutathione transferases in *Cryptosporidium* species. Sci. Rep..

[20] Ingmer H., Vogensen F.K., Hammer K., Kilstrup M. (1999). Disruption and analysis of the *clpB*, *clpC*, and *clpE* genes in *Lactococcus lactis*: ClpE, a new Clp family in gram-positive bacteria. J. Bacteriol..

[21] Lee S., Sowa M.E., Watanabe Y.-H., Sigler P.B., Chiu W., Yoshida M., Tsai F.T. (2003). The structure of ClpB: A molecular chaperone that rescues proteins from an aggregated state. Cell.

[22] Pietrosiuk A., Lenherr E.D., Falk S., Bönemann G., Kopp J., Zentgraf H., Sinning I., Mogk A. (2011). Molecular basis for the unique role of the AAA+ chaperone ClpV in type VI protein secretion. J. Biol. Chem..

[23] Zheng B., Halperin T., Hruskova-Heidingsfeldova O., Adam Z., Clarke A.K. (2002). Characterization of chloroplast Clp proteins in *Arabidopsis*: Localization, tissue specificity and stress responses. Physiol. Plant..

[24] Thibault G., Tsitrin Y., Davidson T., Gribun A., Houry W.A. (2006). Large nucleotide-dependent movement of the N-terminal domain of the ClpX chaperone. EMBO J..

[25] Ali M.S., Baek K.-H. (2020). Protective Roles of Cytosolic and Plastidal Proteasomes on Abiotic Stress and Pathogen Invasion. Plants.

[26] Capestany C.A., Tribble G.D., Maeda K., Demuth D.R., Lamont R.J. (2008). Role of the Clp system in stress tolerance, biofilm formation, and intracellular invasion in *Porphyromonas gingivalis*. J. Bacteriol..

[27] Wojtyra U.A., Thibault G., Tuite A., Houry W.A. (2003). The N-terminal zinc binding domain of ClpX is a dimerization domain that modulates the chaperone function. J. Biol. Chem..

[28] Pavlopoulos G.A., Soldatos T.G., Barbosa-Silva A., Schneider R. (2010). A reference guide for tree analysis and visualization. BioData Min..

[29] Newell P., Fricker A., Roco C., Chandrangsu P., Merkel S. (2013). A Small-Group Activity Introducing the Use and Interpretation of BLAST. J. Microbiol. Biol. Educ. JMBE.

[30] Berg J.M., Tymoczko J.L., Stryer L. (2002). Section 7.1, Homologs Are Descended from a Common Ancestor. Biochemistry.

[31] Wlodawer A., Minor W., Dauter Z., Jaskolski M. (2008). Protein crystallography for non-crystallographers, or how to get the best (but not more) from published macromolecular structures. FEBS J..

[32] Di Tommaso P., Moretti S., Xenarios I., Orobitg M., Montanyola A., Chang J.-M., Taly J.-F., Notredame C. (2011). T-Coffee: A web server for the multiple sequence alignment of protein and RNA sequences using structural information and homology extension. Nucleic Acids Res..

[33] Carugo O., Pongor S. (2001). A normalized root-mean-square distance for comparing protein three-dimensional structures. Protein Sci..

[34] Zhao Y., Zeng C., Massiah M.A. (2015). Molecular Dynamics Simulation Reveals Insights into the Mechanism of Unfolding by the A130T/V Mutations within the MID1 Zinc-Binding Bbox1 Domain. PLoS ONE.

[35] Ko J., Park H., Heo L., Seok C. (2012). GalaxyWEB server for protein structure prediction and refinement. Nucleic Acids Res..

[36] Schrodinger L. (2010). The PyMOL Molecular Graphics System.

[37] Rosenzweig R., Farber P., Velyvis A., Rennella E., Latham M.P., Kay L.E. (2015). ClpB N-terminal domain plays a regulatory role in protein disaggregation. Proc. Natl. Acad. Sci. USA.

[38] Ye J., Osborne A.R., Groll M., Rapoport T.A. (2004). RecA-like motor ATPases—Lessons from structures. Biochim. Biophys. Acta.

[39] Tiwari G., Mohanty D. (2013). An In Silico Analysis of the Binding Modes and Binding Affinities of Small Molecule Modulators of PDZ-Peptide Interactions. PLoS ONE.

[40] Buchner J. (2019). Molecular chaperones and protein quality control: An introduction to the JBC Reviews thematic series. J. Biol. Chem..

[41] Pathak R., Gupta A., Shukla R., Baunthiyal M. (2018). Identification of new drug-like compounds from millets as Xanthine oxidoreductase inhibitors for treatment of Hyperuricemia: A molecular docking and simulation study. Comput. Biol. Chem..

[42] Hashemzadeh H., Javadi H., Darvishi M. (2020). Study of Structural stability and formation mechanisms in DSPC and DPSM liposomes: A coarse-grained molecular dynamics simulation. Sci. Rep..

[43] Deng X., Eickholt J., Cheng J. (2012). A comprehensive overview of computational protein disorder prediction methods. Mol. BioSyst..

[44] Coşkun O. (2016). Separation techniques: Chromatography. North. Clin. Istanb..

[45] Whitfield C.D., Steers E.J., Weissbach H. (1970). Purification and properties of 5-methyltetrahydropteroyltriglutamate-homocysteine transmethylase. J. Biol. Chem..

[46] Bradford M.M. (1976). A rapid and sensitive method for the quantitation of microgram quantities of protein utilizing the principle of protein-dye binding. Anal. Biochem..

[47] Greenfield N.J. (2006). Using circular dichroism spectra to estimate protein secondary structure. Nat. Protoc..

[48] Gasymov O.K., Glasgow B.J. (2007). ANS fluorescence: Potential to augment the identification of the external binding sites of proteins. Biochim. Biophys. Acta.

[49] Aranovich A., Gdalevsky G., Cohen-Luria R., Fishov I., Parola A. (2006). Membrane-catalyzed nucleotide exchange on DnaA—Effect of surface molecular crowding. J. Biol. Chem..

[50] Ngcobo N.S., Chiliza Z.E., Chen W., Yu J.-H., Nelson D.R., Tuszynski J.A., Preto J., Syed K. (2020). Comparative Analysis, Structural Insights, and Substrate/Drug Interaction of CYP128A1 in *Mycobacterium tuberculosis*. Int. J. Mol. Sci..

[51] Letunic I., Bork P. (2019). Interactive Tree of Life (iTOL) v4: Recent updates and new developments. Nucleic Acids Res..

[52] Boc A., Diallo A.B., Makarenkov V. (2012). T-REX: A web server for inferring, validating and visualizing phylogenetic trees and networks. Nucleic Acids Res..

[53] Mullins J.G. (2012). Structural modelling pipelines in next generation sequencing projects. Adv. Protein Chem. Struct. Biol..

[54] Laemmli U.K. (1970). Cleavage of Structural Proteins during the Assembly of the Head of Bacteriophage T4. Nature.

[55] Kelley L.A., Mezulis S., Yates C.M., Wass M.N., Sternberg M.J.E. (2015). The Phyre2 web portal for protein modeling, prediction and analysis. Nat. Protoc..

[56] Yang J., Yan R., Roy A., Xu D., Poisson J., Zhang Y. (2015). The I-TASSER Suite: Protein structure and function prediction. Nat. Methods.

[57] Roy A., Kucukural A., Zhang Y. (2010). I-TASSER: A unified platform for automated protein structure and function prediction. Nat. Protoc..

[58] Zhang Y. (2008). I-TASSER server for protein 3D structure prediction. BMC Bioinform..

[59] Camacho C., Coulouris G., Avagyan V., Ma N., Papadopoulos J., Bealer K., Madden T.L. (2009). BLAST+: Architecture and applications. BMC Bioinform..

[60] Laskowski R.A., MacArthur M.W., Moss D.S., Thornton J.M. (1993). PROCHECK: A program to check the stereochemical quality of protein structures. J. Appl. Crystallogr..

[61] Williams C.J., Headd J.J., Moriarty N.W., Prisant M.G., Videau L.L., Deis L.N., Verma V., Keedy D.A., Hintze B.J., Chen V.B. (2018). MolProbity: More and better reference data for improved all-atom structure validation. Protein Sci..

[62] Schrödinger (2021). Release 2021-2: Maestro.

[63] Sievers F., Wilm A., Dineen D., Gibson T.J., Karplus K., Li W., Lopez R., McWilliam H., Remmert M., Söding J. (2011). Fast, scalable generation of high-quality protein multiple sequence alignments using Clustal Omega. Mol. Syst. Biol..

[64] Schöning-Stierand K., Diedrich K., Fährrolfes R., Flachsenberg F., Meyder A., Nittinger E., Steinegger R., Rarey M. (2020). ProteinsPlus: Interactive analysis of protein–ligand binding interfaces. Nucleic Acids Res..

[65] Mészáros B., Erdős G., Dosztányi Z. (2018). IUPred2A: Context-dependent prediction of protein disorder as a function of redox state and protein binding. Nucleic Acids Res..

[66] Lobley A., Whitmore L., Wallace B.A. (2002). DICHROWEB: An interactive website for the analysis of protein secondary structure from circular dichroism spectra. Bioinformatics.

[67] Kruger N. (1994). The Bradford Method for Protein Quantitation. Methods Mol. Biol..

