# Supplementary material for: Caseinolytic Proteins (Clp) in the Genus Klebsiella: Special Focus on ClpK
Source: Molecules. 2021 Dec 29;27(1):200. doi: 10.3390/molecules27010200 (PMC8746953; doi:10.3390/molecules27010200)
Supplement: Supplementary file 1 [file molecules-27-00200-s001.zip › molecules-1364474-supplementary.pdf]

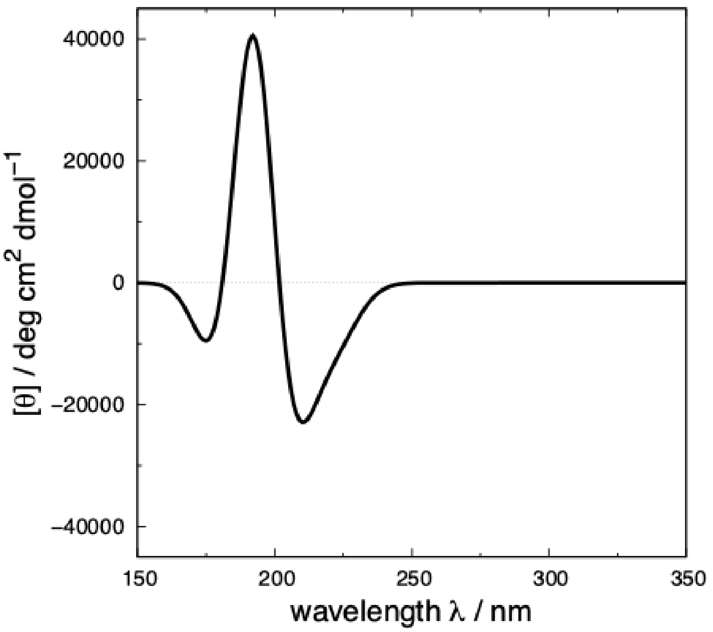

Figure S1: Virtual Circular Dichromism (CD) spectrum of ClpK. The primary amino acid sequence of ClpK was passed through the DichroCalc server, the data obtained from the server was then analysed to understand the secondary structures of ClpK (https://comp.chem.nottingham.ac.uk/dichrocalc/).

Table S1: Ramachandran analysis of the template ClpB and modelled trimeric ClpK protein obtained from ProCheck.

Residues in	Ramachandran statistics	
	1QVR	KpClpK Model
Most favoured regions	83.10%	90.10%
Additional allowed regions	15.20%	8.30%
Generously allowed regions	1.70%	1.20%
Disallowed regions	0.00%	0.40%
Total residues	717	2100

Table S2: DichroWeb[1] analysis of the secondary structure of ClpK.

	Helix (%)	Strand (%)	Turns (%)	Unordered (%)	NRMSD[3]
**ClpK** (952[2])	35.3	22.9	20.7	21	0.12

[1] [1]
[2] Number of amino acids
[3] Normalised root mean square deviation (NRMSD) value is independent of dimensions, and a value of <0.1 is considered acceptable.

Table S3: Properties of the binding pockets in the N-terminal region of ClpB and ClpK identified using DoGSiteScorer[1].

	ClpB	ClpK
**Volume (Å^3)**	1767.82	1511.88
**Surface (Å^2)**	1967.01	1626.42
**Depth (Å)**	31.30	20.80
**Hydrophobicity ratio**	0.34	0.28
**Enclosure**	0.08	0.07
**Frequent amino acid**	Leucine	Glutamine

[1] [2]

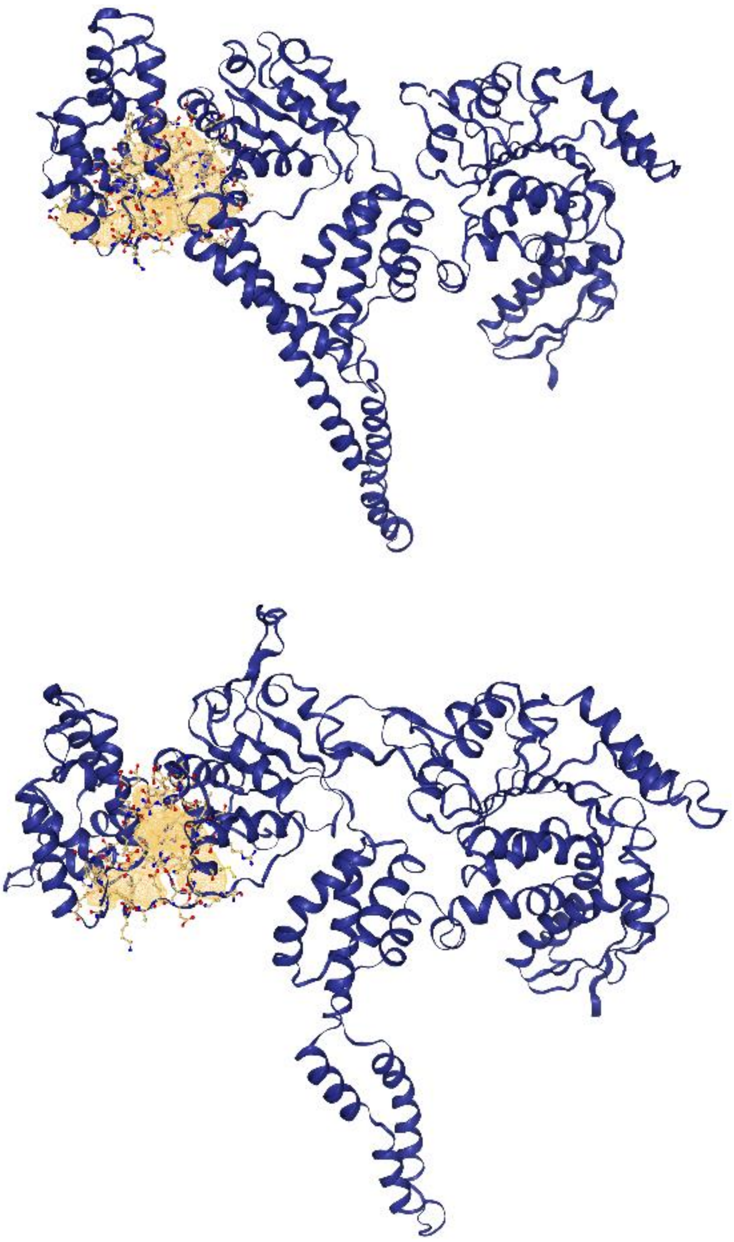

Figure S2: N-terminal binding groove identified in ClpK and ClpB. Top) ClpB with the binding groove shown in yellow. **Bottom)** ClpK with the binding groove identified in yellow. The binding grooves were identified using DoGSiteScorer [2].

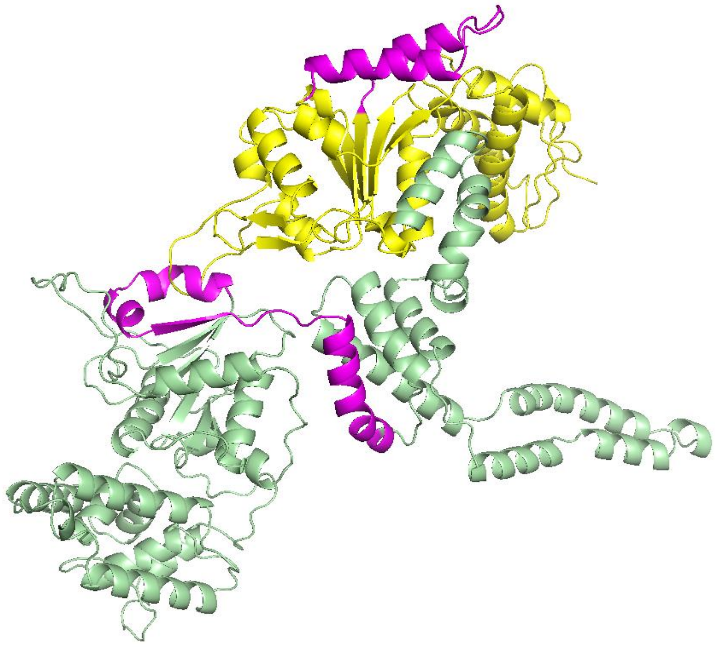

Figure S3: ClpK adopts a RecA-like fold in NBD1 and NBD2. The secondary structural features are coloured as follows; the N-terminal domain and the C-terminal domain are shown as pale green and yellow, respectively. The RecA-like domains are shown in magenta. The protein was visualised using PyMol [3].

```
MARKQCQVCGQPATVRVEANLNGRNSTMLLCDDHYRQLVRQQKRTVSPLEALFGSRSGLF
EDFLGSDFFRIGEDATPVAADTDDVVDASFGEPATAGSGAPRRRGSGLASRISEQSEALL
QEAAKHAAEFGRSEVDTEHLLLALADSDVVKTILGQFKIKVDDLKRQIESEAKRGDKPFE
GEIGVSPRVKDALSRAFVASNELGHSYVGPEHFLIGLAEEGEGLAANLLRRYGLTPQALR
QQVSKVVGKGAEDGRAETPTNTPELDKYSRDLTKMARDGKLDPVIGRAQEIETTIEVLAR
RKKNNPVLIGEPGVGKTAIVEGLAQRMVAGEVPETLRDKRLVELNINAMVAGAKYRGEFE
ERVQKVLKEVTEHQGELILFIDEVHTIVGAGQGGGEGGLDVANVFKPMMARGELNLIGAT
TLNEYQKYIEKDAALERRFQPVTVPEPTVAQAIMILRGLRDTFEAHHKVSISEDAIIAAA
ELSDRYITARFLPDKAIDLLDQAAARVKLSATARPGAVQELESELHQLRREQDYVAARKQ
YDQAAELGKRIEAKEAELKKLVEDWERERASGSAEVKAEHVAQIVSRLTGIPVNELTVEE
REKLLHLEQRLHERLVGQDEAVRAVADAVRLSRAGLREGGKPVATFLFLGPTGVGKTELA
KALAESIYGDEHALLRIDMSEYGERHTVARLVGAPPGYVGYDEGGQLTEKVRRKPYSVLL
LDEIEKAHPDVYNILLQVFDDGRLTDGKGRVVDFTNTIIIATSNLGSDIIQRRLKAREAA
GEEYEKTKAEVMDVLRGHFRPEFLNRIDEIIVFHALHALGKEEIRHIVGLQLDRVARSAA
SQGVTLTFDQTLIDHFAEEGYKPEFGARELKRLIRSELETALAREMLGGGIGKGDHASAR
WDDKAERVVFERKEPPQTPAEPEQPDVAKATETPHGDAGKDSSKKKSASDAS
```

Figure S4: Primary amino acid sequence of ClpK. The sequence was obtained from GenBank (Uniprot: E0W6V3).

Table S3: *Klebsiella* species and strains used for the genomic analysis of Clp proteins. The species name, web-link and references were obtained from the NCBI genomes database for prokaryotes.

Strain	Web-link	References
	K. aerogenes	
CAV1320	https://www.ncbi.nlm.nih.gov/genome/3417?genome_assembly_id=232549	[4, 5]
FDAARGOS_139	https://www.ncbi.nlm.nih.gov/genome/327?genome_assembly_id=363711	[6]
G7	https://www.ncbi.nlm.nih.gov/genome/327?genome_assembly_id=271718	[7]
FDAARGOS_327	https://www.ncbi.nlm.nih.gov/genome/327?genome_assembly_id=1552	[6]

FDAARGOS_152	https://www.ncbi.nlm.nih.gov/genome/327?genome_assembly_id=363028	[6]
FDAARGOS_513	https://www.ncbi.nlm.nih.gov/genome/327?genome_assembly_id=3379	[6]
FDAARGOS_363	https://www.ncbi.nlm.nih.gov/genome/327?genome_assembly_id=34818	[6]
FDAARGOS_641	https://www.ncbi.nlm.nih.gov/genome/3417?genome_assembly_id=695478	[6]
	K. michiganensis	
FDAARGOS_647	https://www.ncbi.nlm.nih.gov/genome/33882?genome_assembly_id=695495	[6]
	K. oxytoca	
CAV1015	https://www.ncbi.nlm.nih.gov/genome/1165?genome_assembly_id=291700	[4, 5]
KONIH5	https://www.ncbi.nlm.nih.gov/genome/1165?genome_assembly_id=361932	[8]
CAV1374	https://www.ncbi.nlm.nih.gov/genome/1165?genome_assembly_id=232487	[4, 5]
CAV1335	https://www.ncbi.nlm.nih.gov/genome/1165?genome_assembly_id=232486	[4, 5]
FDAARGOS_335	https://www.ncbi.nlm.nih.gov/genome/1165?genome_assembly_id=366590	[6]
CAV1752	https://www.ncbi.nlm.nih.gov/genome/1165?genome_assembly_id=303753	[4, 5]
FDAARGOS_500	https://www.ncbi.nlm.nih.gov/genome/1165?genome_assembly_id=3369	[6]
	K. pneumoniae	
2_GR_12	https://www.ncbi.nlm.nih.gov/genome/815?genome_assembly_id=56801	[9, 10]
WCHKP649	https://www.ncbi.nlm.nih.gov/genome/815?genome_assembly_id=375331	[11]
FDAARGOS_775	https://www.ncbi.nlm.nih.gov/genome/815?genome_assembly_id=589914	[6]
TVGHCRE225	https://www.ncbi.nlm.nih.gov/genome/815?genome_assembly_id=380774	[12]

BK13043	https://www.ncbi.nlm.nih.gov/genome/815?genome_assembly_id=319029	[13, 14]
KP64 DNA	https://www.ncbi.nlm.nih.gov/genome/815?genome_assembly_id=16976	[15]
KPNIH48#	https://www.ncbi.nlm.nih.gov/genome/815?genome_assembly_id=363608	[8]
C2660	https://www.ncbi.nlm.nih.gov/genome/815?genome_assembly_id=77054	[16]
WCHKP020037	https://www.ncbi.nlm.nih.gov/genome/815?genome_assembly_id=77722	[11]
GSU10-3 DNA	https://www.ncbi.nlm.nih.gov/genome/815?genome_assembly_id=17291	[17-19]
WCHKP2	https://www.ncbi.nlm.nih.gov/genome/815?genome_assembly_id=382097	[11]
C789	https://www.ncbi.nlm.nih.gov/genome/815?genome_assembly_id=74186	[20]
Isolate blood sample 2	https://www.ncbi.nlm.nih.gov/genome/815?genome_assembly_id=280776	[21]
WCHKP115068	https://www.ncbi.nlm.nih.gov/genome/815?genome_assembly_id=65659	[11]
WCHKP2080	https://www.ncbi.nlm.nih.gov/genome/815?genome_assembly_id=65653	[11]
WCHKP36	https://www.ncbi.nlm.nih.gov/genome/815?genome_assembly_id=373304	[11]
WCHKP020098	https://www.ncbi.nlm.nih.gov/genome/815?genome_assembly_id=65657	[11]
Xen39	https://www.ncbi.nlm.nih.gov/genome/815?genome_assembly_id=786792	[22]
WCHKP115069	https://www.ncbi.nlm.nih.gov/genome/815?genome_assembly_id=27777	[11]
16_GR_13	https://www.ncbi.nlm.nih.gov/genome/815?genome_assembly_id=4137	[9, 10]
FDAARGOS_531	https://www.ncbi.nlm.nih.gov/genome/815?genome_assembly_id=3364	[6]

SCKP020003	https://www.ncbi.nlm.nih.gov/genome/815?genome_assembly_id=10198	[11]
KP617	https://www.ncbi.nlm.nih.gov/genome/815?genome_assembly_id=249502	[23]
XH209#	https://www.ncbi.nlm.nih.gov/genome/815?genome_assembly_id=212989	[24]
CAV1217	https://www.ncbi.nlm.nih.gov/genome/815?genome_assembly_id=296907	[1,2]
1_GR_13	https://www.ncbi.nlm.nih.gov/genome/815?genome_assembly_id=1578	[9, 10]
121	https://www.ncbi.nlm.nih.gov/genome/815?genome_assembly_id=493866	[25]
SCKP020135	https://www.ncbi.nlm.nih.gov/genome/815?genome_assembly_id=476671	[11]
WCHKP095845	https://www.ncbi.nlm.nih.gov/genome/815?genome_assembly_id=13002	[11]
2-1	https://www.ncbi.nlm.nih.gov/genome/815?genome_assembly_id=19	[26]
KP33 DNA	https://www.ncbi.nlm.nih.gov/genome/815?genome_assembly_id=16975	[15]
SCKP020049	https://www.ncbi.nlm.nih.gov/genome/815?genome_assembly_id=20206	[11]
FDAARGOS_630	https://www.ncbi.nlm.nih.gov/genome/815?genome_assembly_id=6954	[6]
FDAARGOS_156	https://www.ncbi.nlm.nih.gov/genome/815?genome_assembly_id=36549	[6]
CFSAN054111	https://www.ncbi.nlm.nih.gov/genome/815?genome_assembly_id=370067	[27-31]
J1	https://www.ncbi.nlm.nih.gov/genome/815?genome_assembly_id=260523	[32, 33]
KpN01	https://www.ncbi.nlm.nih.gov/genome/815?genome_assembly_id=27223	[34]
FDAARGOS_566	https://www.ncbi.nlm.nih.gov/genome/815?genome_assembly_id=3363	[6]

Carbapenem-resistant blaNDM-1	https://www.ncbi.nlm.nih.gov/genome/815?genome_assembly_id=205032	[35]
WCHKP34	https://www.ncbi.nlm.nih.gov/genome/815?genome_assembly_id=382098	[11]
FDAARGOS_631	https://www.ncbi.nlm.nih.gov/genome/815?genome_assembly_id=6953	[3]
TA6363	https://www.ncbi.nlm.nih.gov/genome/815?genome_assembly_id=771384	[6]
CRKP I	https://www.ncbi.nlm.nih.gov/genome/815?genome_assembly_id=521650	[36]
PMK1	https://www.ncbi.nlm.nih.gov/genome/815?genome_assembly_id=211364	[37]
1050	https://www.ncbi.nlm.nih.gov/genome/815?genome_assembly_id=10329	[38]
KPNIH39	https://www.ncbi.nlm.nih.gov/genome/815?genome_assembly_id=277271	[39]
KPN1482	https://www.ncbi.nlm.nih.gov/genome/815?genome_assembly_id=319030	[13, 14]
CAV1392	https://www.ncbi.nlm.nih.gov/genome/815?genome_assembly_id=232380	[5]
KPNIH45	https://www.ncbi.nlm.nih.gov/genome/815?genome_assembly_id=468513	[8]
CAV1344	https://www.ncbi.nlm.nih.gov/genome/815?genome_assembly_id=232381	[4, 5]
CAV1042	https://www.ncbi.nlm.nih.gov/genome/815?genome_assembly_id=296901	[4, 5]
C2414	https://www.ncbi.nlm.nih.gov/genome/815?genome_assembly_id=770443	[16]
39427	https://www.ncbi.nlm.nih.gov/genome/815?genome_assembly_id=908959	[40]
CAV1217	https://www.ncbi.nlm.nih.gov/genome/815?genome_assembly_id=296238	[4, 5]
CAV1453	https://www.ncbi.nlm.nih.gov/genome/815?genome_assembly_id=296230	[4, 5]

FDAARGOS_439	https://www.ncbi.nlm.nih.gov/genome/815?genome_assembly_id=343753	[6]
FDAARGOS_439	https://www.ncbi.nlm.nih.gov/genome/815?genome_assembly_id=343751	[6]
	K pneumoniae* subsp. *pneumoniae	
KPNIH32	https://www.ncbi.nlm.nih.gov/genome/815?genome_assembly_id=21262	[41]
WCHKP015093	https://www.ncbi.nlm.nih.gov/genome/815?genome_assembly_id=65655	[11]
TGH8	https://www.ncbi.nlm.nih.gov/genome/815?genome_assembly_id=272815	[42-44]
KPNIH31	https://www.ncbi.nlm.nih.gov/genome/815?genome_assembly_id=213199	[41]
234-12	https://www.ncbi.nlm.nih.gov/genome/815?genome_assembly_id=230300	[45]
KPNIH30	https://www.ncbi.nlm.nih.gov/genome/815?genome_assembly_id=213198	[41]
KPNIH29	https://www.ncbi.nlm.nih.gov/genome/815?genome_assembly_id=213197	[41]
KPNIH33	https://www.ncbi.nlm.nih.gov/genome/815?genome_assembly_id=212640	[41]
SCKP020079	https://www.ncbi.nlm.nih.gov/genome/815?genome_assembly_id=377125	[11]
SCKP020046	https://www.ncbi.nlm.nih.gov/genome/815?genome_assembly_id=373305	[11]
SCKP040074	https://www.ncbi.nlm.nih.gov/genome/815?genome_assembly_id=377126	[11]
ST:101960100	https://www.ncbi.nlm.nih.gov/genome/815?genome_assembly_id=354684	[46]
TGH10[1]	https://www.ncbi.nlm.nih.gov/genome/815?genome_assembly_id=272816	[42-44]
TGH13	https://www.ncbi.nlm.nih.gov/genome/815?genome_assembly_id=283648	[42-44]
	K. quasipneumoniae	

ATCC 700603	https://www.ncbi.nlm.nih.gov/genome/38419?genome_assembly_id=294621	[9, 10]
HKUOPLA	https://www.ncbi.nlm.nih.gov/genome/3829?genome_assembly_id=20687	[47]
FDAARGOS_93	https://www.ncbi.nlm.nih.gov/genome/3829?genome_assembly_id=36525	[6]
	K. variicola	
WCHKP19	https://www.ncbi.nlm.nih.gov/genome/2121?genome_assembly_id=666503	[47]
DX120E	https://www.ncbi.nlm.nih.gov/genome/2121?genome_assembly_id=280598	[48]
FDAARGOS_627	https://www.ncbi.nlm.nih.gov/genome/2121?genome_assembly_id=695475	[6]
GJ1	https://www.ncbi.nlm.nih.gov/genome/2121?genome_assembly_id=30584	[49]
GJ3	https://www.ncbi.nlm.nih.gov/genome/2121?genome_assembly_id=30586	[49]
KP5-1	https://www.ncbi.nlm.nih.gov/genome/2121?genome_assembly_id=20979	[50]
KPN1481	https://www.ncbi.nlm.nih.gov/genome/2121?genome_assembly_id=20630	[13, 14]
342	https://www.ncbi.nlm.nih.gov/genome/2121?genome_assembly_id=410978	[51]
WCHKV030666	https://www.ncbi.nlm.nih.gov/genome/2121?genome_assembly_id=666504	[11]

References

1. Lobley, A., L. Whitmore, and B.A. Wallace, *DICHROWEB: an interactive website for the analysis of protein secondary structure from circular dichroism spectra.* Bioinformatics, 2002. **18**(1): p. 211-212.
2. Schöning-Stierand, K., et al., *ProteinsPlus: interactive analysis of protein–ligand binding interfaces.* Nucleic Acids Research, 2020. **48**(W1): p. W48-W53.
3. Schrodinger, L., *The PyMOL molecular graphics system.* Version 2010. **, 1, (5), 0.**
4. Mathers, A.J., et al., *Klebsiella pneumoniae carbapenemase (KPC)-producing K. pneumoniae at a single institution: insights into endemicity from whole-genome sequencing.* Antimicrobial agents and chemotherapy, 2015. **59**(3): p. 1656-1663.
5. Sheppard, A.E., et al., *Complete genome sequence of KPC-producing Klebsiella pneumoniae strain CAV1193.* Genome announcements, 2016. **4**(1).
6. Sichtig, H., et al., *FDA-ARGOS is a database with public quality-controlled reference genomes for diagnostic use and regulatory science.* Nature communications, 2019. **10**(1): p. 1-13.
7. Philippe, N., et al., *In vivo evolution of bacterial resistance in two cases of Enterobacter aerogenes infections during treatment with imipenem.* PloS one, 2015. **10**(9): p. e0138828.

8. Weingarten, R.A., et al., *Genomic analysis of hospital plumbing reveals diverse reservoir of bacterial plasmids conferring carbapenem resistance.* MBio, 2018. **9**(1).
9. Elliott, A.G., et al., *Complete genome sequence of Klebsiella quasipneumoniae subsp. similipneumoniae strain ATCC 700603.* Genome announcements, 2016. **4**(3).
10. Pitt, M.E., et al., *Multifactorial chromosomal variants regulate polymyxin resistance in extensively drug-resistant Klebsiella pneumoniae.* Microbial genomics, 2018. **4**(3).
11. Hu, Y., et al., *Klebsiella huaxiensis sp. nov., recovered from human urine.* International journal of systematic and evolutionary microbiology, 2019. **69**(2): p. 333-336.
12. Huang, Y.-H., et al., *Emergence of an XDR and carbapenemase-producing hypervirulent Klebsiella pneumoniae strain in Taiwan.* Journal of Antimicrobial Chemotherapy, 2018. **73**(8): p. 2039-2046.
13. Long, S.W., et al., *Whole-genome sequencing of human clinical Klebsiella pneumoniae isolates reveals misidentification and misunderstandings of Klebsiella pneumoniae, Klebsiella variicola, and Klebsiella quasipneumoniae.* Msphere, 2017. **2**(4).
14. Long, S.W., et al., *Population genomic analysis of 1,777 extended-spectrum beta-lactamase-producing Klebsiella pneumoniae isolates, Houston, Texas: unexpected abundance of clonal group 307.* MBio, 2017. **8**(3).
15. Sakamoto, N., et al., *Genomic characterization of carbapenemase-producing Klebsiella pneumoniae with chromosomally carried blaNDM-1.* Antimicrobial agents and chemotherapy, 2018. **62**(12).
16. Gao, H., et al., *The transferability and evolution of NDM-1 and KPC-2 co-producing Klebsiella pneumoniae from clinical settings.* EBioMedicine, 2020. **51**: p. 102599.
17. Sekizuka, T., et al., *Potential KPC‐2 carbapenemase reservoir of environmental Aeromonas hydrophila and Aeromonas caviae isolates from the effluent of an urban wastewater treatment plant in Japan.* Environmental microbiology reports, 2019. **11**(4): p. 589-597.
18. Sekizuka, T., et al., *Characterization of NDM-5-and CTX-M-55-coproducing Escherichia coli GSH8M-2 isolated from the effluent of a wastewater treatment plant in Tokyo Bay.* Infection and drug resistance, 2019. **12**: p. 2243.
19. Sekizuka, T., et al., *Complete genome sequence of a blaKPC-2-positive Klebsiella pneumoniae strain isolated from the effluent of an urban sewage treatment plant in Japan.* Msphere, 2018. **3**(5).
20. Zhang, Y., et al., *Evolution of hypervirulence in carbapenem-resistant Klebsiella pneumoniae in China: a multicentre, molecular epidemiological analysis.* Journal of Antimicrobial Chemotherapy, 2020. **75**(2): p. 327-336.
21. Babouee, B., et al., *Emergence of four cases of KPC-2 and KPC-3-carrying Klebsiella pneumoniae introduced to Switzerland, 2009–10.* Eurosurveillance, 2011. **16**(11): p. 19817.
22. Singer, J.R., et al., *Preventing dysbiosis of the neonatal mouse intestinal microbiome protects against late-onset sepsis.* Nature medicine, 2019. **25**(11): p. 1772-1782.
23. Kwon, T., et al., *Complete genome sequence of Klebsiella pneumoniae subsp. pneumoniae KP617, coproducing OXA-232 and NDM-1 carbapenemases, isolated in South Korea.* Genome announcements, 2016. **4**(1).
24. Hua, X., et al., *Complete genome sequence of Klebsiella pneumoniae sequence type 17, a multidrug-resistant strain isolated during tigecycline treatment.* Genome announcements, 2014. **2**(6).
25. Ruan, Z., et al., *Emergence of a ST2570 Klebsiella pneumoniae isolate carrying mcr-1 and blaCTX-M-14 recovered from a bloodstream infection in China.* Clinical Microbiology and Infection, 2019. **25**(7): p. 916-918.
26. Li, L., et al., *The genetic structures of an extensively drug resistant (XDR) Klebsiella pneumoniae and its plasmids.* Frontiers in cellular and infection microbiology, 2019. **8**: p. 446.
27. Bjornsdottir-Butler, K., et al., *Draft genome sequences of histamine-producing Photobacterium kishitanii and Photobacterium angustum, isolated from albacore (Thunnus alalunga) and yellowfin (Thunnus albacares) tuna.* Genome announcements, 2015. **3**(2).
28. Pirone-Davies, C., et al., *Genes significantly associated with lineage II food isolates of Listeria monocytogenes.* BMC genomics, 2018. **19**(1): p. 1-11.
29. Pettengill, E.A., et al., *Complete genome sequence of enteroinvasive Escherichia coli O96: H19 associated with a severe foodborne outbreak.* Genome announcements, 2015. **3**(4).
30. Gonzalez-Escalona, N., et al., *Draft genome sequence of bivalent Clostridium botulinum strain IBCA10-7060, encoding botulinum neurotoxin B and a new FA mosaic type.* Genome announcements, 2014. **2**(6).
31. Burall, L.S., et al., *Whole-genome sequencing identifies an atypical Listeria monocytogenes strain isolated from pet foods.* Genome announcements, 2014. **2**(6).
32. Wei, W., et al., *Synergetic effects and flocculation behavior of anionic polyacrylamide and extracellular polymeric substrates extracted from Klebsiella sp. J1 on improving soluble cadmium removal.* Bioresource technology, 2015. **175**: p. 34-41.
33. Yang, J., et al., *Competitive adsorption of heavy metals by extracellular polymeric substances extracted from Klebsiella sp. J1.* Bioresource technology, 2015. **196**: p. 533-539.
34. Lynch, T., et al., *Molecular evolution of a Klebsiella pneumoniae ST278 isolate harboring bla NDM-7 and involved in nosocomial transmission.* The Journal of infectious diseases, 2016. **214**(5): p. 798-806.

35. van Duin, D., et al., *Surveillance of carbapenem-resistant Klebsiella pneumoniae: tracking molecular epidemiology and outcomes through a regional network.* Antimicrobial agents and chemotherapy, 2014. **58**(7): p. 4035-4041.
36. Paul, M., et al., *Genome sequence of a multidrug-resistant Klebsiella pneumoniae ST78 with high colistin resistance isolated from a patient in India.* Journal of global antimicrobial resistance, 2019. **17**: p. 187-188.
37. Stoesser, N., et al., *Genome sequencing of an extended series of NDM-producing Klebsiella pneumoniae isolates from neonatal infections in a Nepali hospital characterizes the extent of community-versus hospital-associated transmission in an endemic setting.* Antimicrobial agents and chemotherapy, 2014. **58**(12): p. 7347-7357.
38. Lázaro-Perona, F., et al., *Genomic path to pandrug resistance in a clinical isolate of Klebsiella pneumoniae.* International journal of antimicrobial agents, 2018. **52**(5): p. 713-718.
39. Conlan, S., et al., *Plasmid Dynamics in KPC-Positive Klebsiella pneumoniae during Long-Term Patient Colonization.* mBio, 2016. **7**(3).
40. Satlin, M.J., et al., *Multicenter clinical and molecular epidemiological analysis of bacteremia due to carbapenem-resistant Enterobacteriaceae (CRE) in the CRE epicenter of the United States.* Antimicrobial agents and chemotherapy, 2017. **61**(4).
41. Conlan, S., et al., *Single-molecule sequencing to track plasmid diversity of hospital-associated carbapenemase-producing Enterobacteriaceae.* Science translational medicine, 2014. **6**(254): p. 254ra126-254ra126.
42. Malhotra-Kumar, S., et al., *Colistin resistance gene mcr-1 harboured on a multidrug resistant plasmid.* The Lancet infectious diseases, 2016. **16**(3): p. 283-284.
43. Ramirez, M.S., et al., *Whole-genome comparative analysis of two carbapenem-resistant ST-258 Klebsiella pneumoniae strains Isolated during a North-eastern Ohio outbreak: Differences within the high heterogeneity zones.* 2016, Oxford University Press.
44. Xavier, B., et al., *Complete sequence of an IncFII plasmid harbouring the colistin resistance gene mcr-1 isolated from Belgian pig farms.* Journal of Antimicrobial Chemotherapy, 2016. **71**(8): p. 2342-2344.
45. Becker, L., et al., *Complete genome sequence of a CTX-M-15-producing Klebsiella pneumoniae outbreak strain from multilocus sequence type 514.* Genome announcements, 2015. **3**(4).
46. Osei Sekyere, J. and D.G. Amoako, *Genomic and phenotypic characterisation of fluoroquinolone resistance mechanisms in Enterobacteriaceae in Durban, South Africa.* PLoS One, 2017. **12**(6): p. e0178888.
47. Lu, M.G.-X., et al., *Complete genome sequence of Klebsiella variicola strain HKUOPLA, a cellulose-degrading bacterium isolated from giant panda feces.* Genome announcements, 2015. **3**(5).
48. Lin, L., et al., *Complete genome sequence of endophytic nitrogen-fixing Klebsiella variicola strain DX120E.* Standards in genomic sciences, 2015. **10**(1): p. 1-7.
49. Di, D.Y., et al., *Emergence of Klebsiella variicola positive for NDM-9, a variant of New Delhi metallo-β-lactamase, in an urban river in South Korea.* Journal of Antimicrobial Chemotherapy, 2017. **72**(4): p. 1063-1067.
50. Medrano, E.G., M.M. Forray, and A.A. Bell, *Complete genome sequence of a Klebsiella pneumoniae strain isolated from a known cotton insect boll vector.* Genome announcements, 2014. **2**(4).
51. Fouts, D.E., et al., *Complete genome sequence of the N 2-fixing broad host range endophyte Klebsiella pneumoniae 342 and virulence predictions verified in mice.* PLoS Genet, 2008. **4**(7): p. e1000141.
